# Emergence of carbapenemase-producing *Escherichia coli* in acute care hospitals in 32 European countries (the CCRE survey): a prospective, multicentre, cross-sectional, epidemiological, microbiological, and genomic surveillance study

**DOI:** 10.1016/j.lanmic.2025.101321

**Published:** 2026-06

**Authors:** Sophia David, Anke Kohlenberg, Corin Yeats, Khalil Abu-Dahab, Barbara Albiger, Nabil-Fareed Alikhan, Erik Alm, Sara Byfors, Natacha Couto, Julio Diaz Caballero, Christian G Giske, Corinna Glasner, Marius Linkevicius, Erika Matuschek, Daniel Palm, Olov Svartström, Hajo Grundmann, Marc J Struelens, Karin Tegmark Wisell, Dominique L Monnet, Inga Fröding, David M Aanensen, Alma Brolund, EURGen-Net CCRE Survey Working Group, Andi Koraqi, Artan Bego, Petra Apfalter, Rainer Hartl, Te-Din Huang, Katrien Latour, Maja Travar, Stefana Sabtcheva, Arjana Tambić-Andrašević, Jaroslav Hrabák, Helena Žemličková, Panayiota Maikanti Charalambous, Anette M Hammerum, Anastasia Bilozor, Marika Jürna-Ellam, Jari Jalava, Kati Räisänen, Laurent Dortet, Ines Noll, Niels Pfennigwerth, Kyriaki Tryfinopoulou, Alkiviadis Vatopoulos, Ákos Tóth, Kristjan Orri Helgason, Martin Cormican, Maria Del Grosso, Maria Giufrè, Arsim Kurti, Lul Raka, Baiba Niedre-Otomere, Jelena Razmuk, Jekaterina Sinotova, Marie Meo, Monique Perrin, Denise Micallef, Nina Nestorova, Milena Lopičić, Vineta Vuksanović, Antoni P A Hendrickx, Karuna E W Vendrik, Ana Kaftandzieva, Dugagjin Osmani, Ørjan Samuelsen, Elżbieta Literacka, Manuela Caniça, Vera Manageiro, Irina Codita, Brandusa Lixandru, Ivana Ćirković, Deana Medić, Milan Nikš, Mateja Pirš, Belén Aracil, Jesús Oteo Iglesias, Petra Edquist, Karin Westmo, Hüsniye Şimşek, Serap Süzük Yildiz, Katie L Hopkins, Danièle Meunier

**Affiliations:** a Centre for Genomic Pathogen Surveillance, Pandemic Sciences Institute, University of Oxford, Oxford, UK; b European Centre for Disease Prevention and Control, Solna, Sweden; c Public Health Agency of Sweden, Solna, Sweden; d Department of Laboratory Medicine, Division of Clinical Microbiology, Karolinska Institute, Stockholm, Sweden; e Department of Clinical Microbiology, Karolinska University Hospital, Stockholm, Sweden; f University of Groningen, Groningen, Netherlands; g European Committee on Antimicrobial Susceptibility Testing Development Laboratory, Växjö, Sweden; h Institute for Infection Prevention and Hospital Epidemiology, Faculty of Medicine, University of Freiburg, Freiburg, Germany; i Faculty of Medicine, Université Libre de Bruxelles, Brussels, Belgium

## Abstract

**Background:**

The emergence of carbapenem resistance in *Escherichia coli* is of major concern due to the high propensity of spread of this species and scarce treatment options. Herein, we examined the occurrence and spread of carbapenem-resistant *E coli* based on the carbapenem-resistant and/or colistin-resistant Enterobacterales (CCRE) survey performed across European countries in 2019.

**Methods:**

We analysed epidemiological, microbiological, and whole-genome sequencing data of 548 *E coli* isolates from individual patients from 156 hospitals in 32 European countries over 6 months in 2019. These hospitals collected the first ten successive isolates of carbapenem-resistant or carbapenem-susceptible increased exposure (carbapenem-R/I) *Klebsiella pneumoniae* species complex or *E coli,* and carbapenem-susceptible (carbapenem-S) comparator isolates of the same species. Antimicrobial susceptibility testing was performed for 19 antimicrobial agents. Whole-genome sequencing was performed centrally using Illumina technology. Isolates from the CCRE survey were compared with those from the European Survey of Carbapenemase-Producing Enterobacteriaceae (EuSCAPE) study.

**Findings:**

Of the 548 *E coli* isolates, 211 (38·5%) were carbapenem-resistant or susceptible, increased exposure (carbapenem-R/I), and 337 (61·5%) were carbapenem-susceptible (carbapenem-S). Five sequence types (STs) accounted for 96 (45·5%) of 211 carbapenem-R/I isolates: ST131 (27), ST410 (20), ST38 (19), ST167 (16), and ST648 (14). Carbapenemase genes were identified in 182 (86·3%) carbapenem-R/I isolates, a pronounced increase from the 2013–14 EuSCAPE study (36 of 99, 36·4%). The most common genes were *bla*_NDM−5_ (62 of 182, 34·1%) and *bla*_OXA−48_ (40 of 182, 22·0%). *bla*_NDM−5_ carriage increased substantially compared with that in EuSCAPE (two of 99, 2·02%). Phylogenetic analysis showed substantial clonal spread of globally disseminated *bla*_NDM−5_-harbouring lineages, with numerous introductions into Europe but minimal onward transmission.

**Interpretation:**

High-risk STs of *E coli* carrying carbapenemase genes are rapidly spreading globally, although our results indicate that, in 2019, most cases in Europe were sporadic. We urge vigilant monitoring, including genomic surveillance, and strengthening of control efforts, to reduce mortality and morbidity associated with the impending rise in carbapenem-R/I *E coli* cases.

**Funding:**

European Centre for Disease Prevention and Control and the Centre for Genomic Pathogen Surveillance.

## Introduction

*Escherichia coli* is the leading cause of deaths attributable to antimicrobial resistance among bacterial pathogens.^1^ The increase in the prevalence of multidrug-resistant *E coli* was initially driven by the global dissemination of high-risk lineages resistant to third-generation cephalosporins and fluoroquinolones (eg, ST131), followed by a rise in resistance to carbapenems.^2^ Both third-generation cephalosporin-resistant and carbapenem-resistant Enterobacterales, which include *E coli*, are recognised by WHO as critical-priority pathogens for the development of new antimicrobial agents.^3^ In Europe, carbapenem resistance emerged and spread more slowly in *E coli* than in *Klebsiella pneumoniae.*^4^ However, there have been increasing reports of carbapenem-resistant *E coli* in the last decade from several regions worldwide, associated with particular lineages and various types of co-resistance.^5^ In addition, the potential spread of plasmid-mediated colistin resistance genes (*mcr* genes) could further reduce treatment options for carbapenem-resistant *E coli*.^6^

**Research in context**

**Evidence before this study**

We searched PubMed for reports published between Jan 1, 2000, and Oct 4, 2025, with no language restrictions and no other exclusion criteria, using the search terms (“carbapenemase-producing” OR “carbapenem-resistant”) AND (“*Escherichia coli*”) AND (“Europe”) AND (“genomic surveillance”). This search identified 83 publications, which mainly consisted of regional or national surveillance studies, outbreak investigations, single-hospital studies, or analyses of the distribution of specific sequence types (STs) or specific resistance genes. No other whole-genome sequencing-based study of carbapenem-resistant or carbapenemase-producing *E coli* with similarly comprehensive European coverage and standardised sampling was identified.

**Added value of this study**

This is the second large-scale structured genomic survey of carbapenem-resistant Enterobacterales coordinated by the European Centre for Disease Prevention and Control, following the European Survey of Carbapenemase-Producing Enterobacteriaceae (EuSCAPE) in 2013–14. We provide epidemiological, microbiological, and whole-genome sequencing data on 548 carbapenem-resistant or susceptible, increased exposure (carbapenem-R/I) and carbapenem-susceptible (carbapenem-S) *E coli* isolates collected in 156 hospitals across 32 countries in 2019. We analyse epidemiological characteristics, report phenotypic susceptibility patterns and associated resistance genes, and provide a reference dataset to which future surveillance data can be compared. We also newly report the *E coli* genomes from the 2013–14 EuSCAPE study, facilitating a temporal comparison of the population structure and genomic characteristics.

We detected one or more carbapenemase genes (or novel related variants) in 182 (86·3%) of 211 carbapenem-R/I isolates from the 2019 carbapenem-resistant and/or colistin-resistant Enterobacterales survey (CCRE survey), representing a pronounced increase from EuSCAPE, in which 36 (36·4%) of 99 carbapenem-R/I isolates from 2013 to 2014 carried a carbapenemase gene. Another key difference between the surveys was the increase in *bla*_NDM-5_ among carbapenem-R/I isolates, from two (2·0%) of 99 isolates in 2013–14 to 62 (29·4%) of 211 isolates in 2019. Our analysis also showed that carbapenem-R/I *E coli* isolates from 2019 were mostly from high-risk STs that are dispersed across Europe. We found evidence for repeated acquisition of different carbapenemase genes in an already dominant lineage, ST131. In addition, there was substantial clonal spread of globally distributed lineages carrying *bla*_NDM-5_ and other carbapenemase genes from ST167, ST361, ST405, ST410, and ST648, although isolates from European countries largely occurred as singletons or in small clusters in the global phylogenies, demonstrating numerous imports into European countries but with so far minimal onward transmission.

**Implications of all the available evidence**

There are various warning signals of a worsening epidemiological situation regarding carbapenem-resistant *E coli* in Europe, relating to the increased acquisition of carbapenemase genes by high-risk STs and increased clonal spread of successful multidrug-resistant lineages. Although carbapenem resistance in *E coli* is still uncommon in Europe, with minimal local transmission identified in 2019, there is a high risk that carbapenem-resistant *E coli* will become endemic in Europe in the absence of further public health action. A rise in carbapenem resistance at a similar rapid pace to that of resistance to third-generation cephalosporins and fluoroquinolones in the past would pose an unprecedented threat to patients with *E coli* infections in Europe and worldwide.

We conducted a survey of carbapenem-resistant and/or colistin-resistant Enterobacterales (CCRE survey) across 37 European countries in 2019 to evaluate the occurrence, geographical distribution, and population dynamics of high-risk lineages and related transmissible resistance elements to inform control measures. Here, we present the CCRE survey findings focused on carbapenem-resistant *E coli* and compare them with the results from the 2013–14 European Survey of Carbapenemase-Producing Enterobacteriaceae (EuSCAPE) study.^7^

## Methods

### Study design and data

The CCRE survey protocol for data and isolate collection is described in an accompanying manuscript,^8^ with detailed methods outlined in epidemiological and microbiological manuals.^9^, ^10^, ^11^ In short, laboratories of sentinel hospitals in 37 countries, including all EU, European Economic Area, and EU candidate countries in 2019, were selected based on geographical and population representativeness. Each recruited hospital microbiology laboratory collected the first ten non-duplicate isolates of carbapenem-resistant or carbapenem-susceptible increased exposure (hereafter called carbapenem-R/I) *K pneumoniae* species complex or *E coli* from clinical or screening samples of consecutive patients. For each carbapenem-R/I isolate, the next available carbapenem-susceptible (carbapenem-S) isolate of the same species was collected as a comparator. Isolate collection started in 2019 with three possible starting dates (ie, March 1, April 1, or May 1, 2019) and ended after collection of ten carbapenem-R/I and carbapenem-S isolates or a maximum period of 6 months. For each isolate, epidemiological patient data as outlined in the protocol^9^ were collected via an online form. All data were pseudonymised and collected in accordance with the European Parliament and Council decisions on the epidemiological surveillance and control of communicable disease in the European community. Ethics approval and informed consent were, thus, not required.

### Procedures

Each participating laboratory performed antimicrobial susceptibility testing (AST) using established routine methods, primarily disk diffusion or broth microdilution, according to European Committee on Antimicrobial Susceptibility Testing (EUCAST) guidelines.^12^ Results of gradient tests and automated methods were also accepted. The recommended panel for testing included 19 antimicrobial agents: ampicillin, amoxicillin–clavulanic acid, aztreonam, piperacillin–tazobactam, cefotaxime, ceftazidime, cefepime, ceftazidime–avibactam, ertapenem, imipenem, meropenem, ciprofloxacin, trimethoprim–sulfamethoxazole, amikacin, gentamicin, tobramycin, colistin, tigecycline, and fosfomycin. Results were interpreted by participating laboratories, with the exception of the results for carbapenems, for which an additional validation was performed. For colistin, only results reported as tested with broth microdilution in line with EUCAST recommendations^12^ were included in the analysis.

EUCAST breakpoint table (version 9), 2019, was used for interpretation of AST results.^12^ *E coli* isolates were included as carbapenem-R/I when one or more of the tested carbapenems were in the resistant (R) or susceptible, increased exposure (I) categories and as carbapenem-S when results were in the susceptible (S) category for all tested carbapenems.

### Whole-genome sequencing (WGS)

WGS was performed centrally using Illumina NovaSeq 6000 (Illumina Inc, San Diego, CA, USA) instruments with 150-base pair paired-end reads. Raw reads were assembled using SPAdes (version 3.10.0) with the “careful” flag provided and the “−cov-cutoff” flag set to “auto”.^13^ Genome assemblies were annotated using Prokka (version 1.5).^14^ Species designations and assembly metrics were obtained using Pathogenwatch (version 12.2.1). Isolates with species designations that were inconsistent with the species identification provided by the National Reference Laboratories (NRLs), and those with 1000 contigs or more or an assembly length outside of 4·5–6·5 Mb, were excluded. *E coli* isolates obtained from the EuSCAPE project (2013–14) were sequenced as described previously^7^ and subjected to the same assembly and quality control procedures described earlier.

Multilocus sequence typing profiles (Warwick scheme) were obtained using Enterobase with new sequence types (STs) defined when necessary.^15^ Replicon typing data were obtained using Pathogenwatch (version 12.2.1) using the PlasmidFinder database (version 2021-11-29).^16^ Resistance genes and mutations were identified using AMRFinderPlus (version 3.10.23).^17^ Inconsistencies in the identification of carbapenemase genes between NRL and WGS results that could not be resolved after a clarification request to the NRL led to the exclusion of isolates. New allele numbers for novel putative carbapenemase variants were assigned by submission to GenBank.

Core genes (present in ≥95% of isolates) were identified among CCRE survey *E coli* isolates using Panaroo (version 1.2.9)^18^ in the “moderate” mode. Single nucleotide polymorphism (SNP) distances were calculated from the core genome alignment using pairsnp. Using the pairwise SNP matrix, we determined the number of SNPs between each isolate and its nearest neighbour from the same hospital, a different hospital in the same country, and a different country. Although the sampling protocol stipulated that isolates should be collected from individual patients, we also removed 55 isolates from the pairwise SNP analysis (but not any other analysis) when duplicate isolates could not be excluded (appendix 2), based on either the absence of patient age or sex information (31 isolates) or matching age and sex profiles among same-hospital isolates (24 isolates).

To construct a phylogenetic tree of the CCRE survey and EuSCAPE *E coli* isolates, core genes were again identified using Panaroo (version 1.2.9). An alignment of the concatenated core genes was used as input to RAxML-NG (version 1.0.3)^19^ for tree construction. Additional phylogenetic trees were constructed for genomes assigned as ST167, ST361, ST405, ST410, and ST648. We included all survey isolates from these STs (and their nested STs), along with public genomes (up to March, 2022) and those from a 2023 report.^20^ Additional CCRE survey isolates that formed closely related outgroups (as determined using the species-wide phylogeny) were included to enable accurate rooting and later removed. Sequence reads were mapped to ST-specific reference genomes: CP074120.1 (ST167), CP083701.1 (ST361), CP090074.1 (ST405), NZ_CP013112.1 (ST410), and CP023815.1 (ST648). Mapping and SNP-calling were performed using Burrows Wheeler Aligner^21^ and SAMtools (version 1.2) mpileup and BCFtools (version 1.2).^22^ Isolates were excluded from downstream analyses when they had less than 20× average mapping depth or greater than or equal to 25% missing sites in the pseudogenome alignment. Gubbins (version 3.0.0)^23^ was used to remove recombined regions and generate maximum likelihood trees with RAxML (version 8.2.12). Microreact^24^ was used for the visualisation of phylogenetic trees with metadata.^19^

### Outcomes

The main outcomes of this study were the distribution of STs and resistance genes of carbapenem-R/I *E coli* circulating in European acute care hospitals in 32 countries in comparison to carbapenem-S comparator isolates from the same hospitals. Epidemiological characteristics and phenotypic AST results of both groups were also evaluated. Comparison of the data from this study to those from the previous EuSCAPE project allowed detection of changes in the composition of STs and resistance genes over time.

### Statistical analysis

Statistical analyses were performed using RStudio (version 4.3.1). For proportions shown in table 1 and appendix 1 p 2, robust standard errors clustered by hospital and country were estimated with the sandwich estimator, and 95% logit transformed Wald confidence intervals were obtained. Missing data were handled through listwise deletion, and the proportions of missing data for each variable are shown in table 1.

**Table 1:** Epidemiological characteristics associated with carbapenem-R/I and carbapenem-S *Escherichia coli* isolates from the carbapenem-resistant and/or colistin-resistant Enterobacterales (CCRE) survey

	Total *E coli* isolates	Carbapenem-R/I *E coli* isolates	Carbapenem-S *E coli* isolates
**Countries**	32	28	29
**Hospitals**	156	127	138
**Isolates**	548	211	337
**Sex**			
Male	246 (44·9%, 40·8–49·1)	119 (56·4%, 49·6–63·0)	127 (37·7%, 32·7–43·0)
Female	273 (49·8%, 45·6–54·0)	84 (39·8%, 33·4–46·6)	189 (56·1%, 50·7–61·3)
Missing	29 (5·3%, 3·7–7·5)	8 (3·8%, 1·9–7·4)	21 (6·2%, 4·1–9·4)
**Age, years**			
0–19	47 (8·6%, 6·5–11·2)	15 (7·1%, 4·3–11·5)	32 (9·5%, 6·8–13·1)
20–39	61 (11·1%, 8·8–14·1)	27 (12·8%, 8·9–18·0)	34 (10·1%, 7·3–13·8)
40–59	103 (18·8%, 15·7–22·3)	40 (19·0%, 14·2–24·8)	63 (18·7%, 14·9–23·2)
60–79	204 (37·2%, 33·3–41·4)	93 (44·1%, 37·5–50·9)	111 (32·9%, 28·1–38·1)
≥80	102 (18·6%, 15·6–22·1)	28 (13·3%, 9·3–18·6)	74 (22·0%, 17·9–26·7)
Missing	31 (5·7%, 4·0–7·9)	8 (3·8%, 1·9–7·4)	23 (6·8%, 4·6–10·1)
**Hospitalisation status**			
Outpatient	129 (23·5%, 20·2–27·3)	44 (20·9%, 15·9–26·9)	85 (25·2%, 20·9–30·1)
Inpatient	389 (71·0%, 67·0–74·6)	154 (73·0%, 66·6–78·6)	235 (69·7%, 64·6–74·4)
Missing	30 (5·5%, 3·9–7·7)	13 (6·2%, 3·6–10·3)	17 (5·0%, 3·2–8·0)
**Type of ward**			
Medical	247 (45·1%, 40·9–49·3)	82 (38·9%, 32·5–45·6)	165 (49·0%, 43·6–54·3)
Intensive care	74 (13·5%, 10·9–16·6)	39 (18·5%, 13·8–24·3)	35 (10·4%, 7·5–14·1)
Surgical	99 (18·1%, 15·1–21·5)	35 (16·6%, 12·1–22·2)	64 (19·0%, 15·1–23·5)
Other	87 (15·9%, 13·0–19·2)	38 (18·0%, 13·4–23·8)	49 (14·5%, 11·2–18·7)
Missing	41 (7·5%, 5·6–10·0)	17 (8·1%, 5·1–12·6)	24 (7·1%, 4·8–10·4)
**Infection and carriage status**			
Carriage	119 (21·7%, 18·5–25·4)	82 (38·9%, 32·5–45·6)	37 (11·0%, 8·1–14·8)
Infection	363 (66·2%, 62·2–70·1)	105 (49·8%, 43·0–56·5)	258 (76·6%, 71·7–80·8)
Undetermined	40 (7·3%, 5·4–9·8)	13 (6·2%, 3·6–10·3)	27 (8·0%, 5·5–11·4)
Missing	26 (4·7%, 3·2–6·9)	11 (5·2%, 2·9–9·2)	15 (4·5%, 2·7–7·3)
**Organ system, location of infection, or carriage**			
Urinary tract	218 (39·8%, 35·8–43·9)	67 (31·8%, 25·8–38·4)	151 (44·8%, 39·6–50·2)
Lower respiratory tract	19 (3·5%, 2·2–5·4)	8 (3·8%, 1·9–7·4)	11 (3·3%, 1·8–5·8)
Intraabdominal	37 (6·8%, 4·9–9·2)	18 (8·5%, 5·4–13·1)	19 (5·6%, 3·6–8·7)
Blood stream	86 (15·7%, 12·9–19·0)	14 (6·6%, 4·0–10·9)	72 (21·4%, 17·3–26·1)
Skin/soft tissue	34 (6·2%, 4·5–8·6)	17 (8·1%, 5·1–12·6)	17 (5·0%, 3·2–8·0)
Other	85 (15·5%, 12·7–18·8)	57 (27·0%, 21·4–33·4)	28 (8·3%, 5·8–11·8)
Missing	69 (12·6%, 10·1–15·6)	30 (14·2%, 10·1–19·6)	39 (11·6%, 8·6–15·5)
**Type of sample**			
Clinical sample	429 (78·3%, 74·6–81·5)	126 (59·7%, 52·9–66·1)	303 (89·9%, 86·2–92·7)
Screening sample	111 (20·3%, 17·1–23·8)	82 (38·9%, 32·5–45·6)	29 (8·6%, 6·0–12·1)
Missing	8 (1·5%, 0·7–2·9)	3 (1·4%, 0·5–4·3)	5 (1·5%, 0·6–3·5)
**Origin of sample**			
Urine	236 (43·1%, 39·0–47·3)	71 (33·6%, 27·6–40·3)	165 (49·0%, 43·6–54·3)
Blood	94 (17·2%, 14·2–20·5)	15 (7·1%, 4·3–11·5)	79 (23·4%, 19·2–28·3)
Wound swabs	31 (5·7%, 4·0–7·9)	17 (8·1%, 5·1–12·6)	14 (4·2%, 2·5–6·9)
Aspirates	18 (3·3%, 2·1–5·2)	5 (2·4%, 1·0–5·6)	13 (3·9%, 2·3–6·5)
Lower respiratory tract specimens	15 (2·7%, 1·7–4·5)	6 (2·8%, 1·3–6·2)	9 (2·7%, 1·4–5·1)
Soft tissue samples	10 (1·8%, 1·0–3·4)	2 (0·9%, 0·2–3·7)	8 (2·4%, 1·2–4·7)
Catheter exit site	6 (1·1%, 0·5–2·4)	4 (1·9%, 0·7–5·0)	2 (0·6%, 0·1–2·3)
Reproductive tract samples	5 (0·9%, 0·4–2·2)	1 (0·5%, 0·1–3·3)	4 (1·2%, 0·4–3·1)
Bone and joint specimens	1 (0·2%, 0·0–1·3)	0	1 (0·3%, 0·0–2·1)
Cerebrospinal fluid	0	0	0
Other	108 (19·7%, 16·6–23·3)	73 (34·6%, 28·5–41·3)	35 (10·4%, 7·5–14·1)
Missing	24 (4·4%, 3·0–6·5)	17 (8·1%, 5·1–12·6)	7 (2·1%, 1·0–4·3)
**Hospital or community acquisition**			
Community-onset	243 (44·3%, 40·2–48·5)	68 (32·2%, 26·3–38·8)	175 (51·9%, 46·6–57·2)
Hospital-acquired	180 (32·8%, 29·0–36·9)	87 (41·2%, 34·8–48·0)	93 (27·6%, 23·1–32·6)
Missing	125 (22·8%, 19·5–26·5)	56 (26·5%, 21·0–32·9)	69 (20·5%, 16·5–25·1)
**Previous hospital admission within 6 months**∗			
Yes	164 (29·9%, 26·2–33·9)	92 (43·6%, 37·0–50·4)	72 (21·4%, 17·3–26·1)
No	166 (30·3%, 26·6–34·3)	34 (16·1%, 11·7–21·7)	132 (39·2%, 34·1–44·5)
Missing	218 (39·8%, 35·8–43·9)	85 (40·3%, 33·9–47·1)	133 (39·5%, 34·4–44·8)
**Previous residence in a long-term care or residential care facility within 6 months**			
Yes	17 (3·1%, 1·9–4·9)	7 (3·3%, 1·6–6·8)	10 (3·0%, 1·6–5·4)
No	235 (42·9%, 38·8–47·1)	84 (39·8%, 33·4–46·6)	151 (44·8%, 39·6–50·2)
Missing	296 (54·0%, 49·8–58·2)	120 (56·9%, 50·1–63·4)	176 (52·2%, 46·9–57·5)
**Previous travel within 6 months**†			
Yes	36 (6·6%, 4·8–9·0)	30 (14·2%, 10·1–19·6)	6 (1·8%, 0·8–3·9)
No reported travel or information not available	512 (93·4%, 91·0–95·2)	181 (85·8%, 80·4–89·9)	331 (98·2%, 96·1–99·2)

Data are n (%, 95% CI). I=susceptible, increased exposure. R=resistant. S=susceptible.

∗ Previous hospital admission within 6 months includes direct transfer from another hospital.

† Previous travel within 6 months includes direct transfer from a hospital in another country and previous hospital admission in another country.

### Role of the funding source

The European Centre for Disease Prevention and Control (ECDC) staff provided input to the study design data collection, data analysis, data interpretation, and writing of the report and managed European Antimicrobial Resistance Genes Surveillance Network (EURGen-Net) and recruitment of national CCRE survey coordinators. The Centre for Genomic Pathogen Surveillance funded the WGS analysis and provided input to the study design, data collection, data analysis, data interpretation, and writing of the report. The decision to submit for publication was jointly taken by representatives of the ECDC, Centre for Genomic Pathogen Surveillance, and The Public Health Agency of Sweden.

## Results

National technical coordinators in 37 European countries recruited 527 hospitals for the CCRE survey, of which 346 (65·7%) hospitals from 36 countries registered *K pneumoniae* species complex or *E coli* isolates over 6 months in 2019. After quality control, the final dataset for *E coli* included 211 carbapenem-R/I and 337 carbapenem-S isolates collected in 156 hospitals in 32 countries (appendix 1 p 5, appendix 2). The number of *E coli* isolates contributed by individual countries was highly variable, ranging from one (Hungary) to 90 isolates (UK; table 2). The CCRE survey collection of Kosovo, Malta, Montenegro, and Slovakia only included *K pneumoniae* but not *E coli* isolates. 24 of the 156 hospitals in the CCRE survey had also contributed *E coli* isolates to the final genomic dataset from EuSCAPE (2013–14), which comprises a total of 221 isolates from 101 hospitals in 26 countries and for which the genomes are newly reported here.

**Table 2:** Carbapenem-R/I (n=211) and carbapenem-S (n=337) *Escherichia coli* isolates and carbapenemase genes by country, collected during the carbapenem-resistant and/or colistin-resistant Enterobacterales (CCRE) survey

	Hospitals	Carbapenem-R/I *E coli* isolates								Carbapenem-S *E coli* isolates	Total *E coli* isolates
		Total carbapenem-R/I *E coli* isolates	*E coli* isolates carrying carbapenemase genes						Isolates without carbapenemase genes		
			Total isolates carrying carbapenemase genes	*Isolates carrying bla*_KPC_	*Isolates carrying bla*_NDM_	*Isolates carrying bla*_OXA−48_−like	*Isolates carrying bla*_VIM_	*Isolates carrying* other or multiple carbapenemase genes			
**Albania**	0	0	0	0	0	0	0	0	0	0	0
**Austria**	4	5	4	0	1	3	0	0	1	5	10
**Belgium**	8	10	9	0	1	8	0	0	1	13	23
**Bosnia and Herzegovina**	1	0	0	0	0	0	0	0	0	3	3
**Bulgaria**	2	7	6	0	5	0	0	1	1	7	14
**Croatia**	1	0	0	0	0	0	0	0	0	4	4
**Cyprus**	1	1	1	1	0	0	0	0	0	1	2
**Czech Republic**	4	10	10	0	9	0	0	1	0	8	18
**Denmark**	4	6	6	0	5	1	0	0	0	0	6
**Estonia**	1	2	1	0	0	0	1	0	1	3	5
**Finland**	5	10	10	0	5	4	0	1	0	0	10
**France**	5	7	7	0	3	4	0	0	0	12	19
**Germany**	12	14	10	0	4	6	0	0	4	21	35
**Greece**	4	2	2	0	0	0	2	0	0	6	8
**Hungary**	1	1	0	0	0	0	0	0	1	0	1
**Iceland**	1	1	1	0	1	0	0	0	0	2	3
**Ireland**	2	1	1	0	1	0	0	0	0	7	8
**Italy**	17	17	17	13	2	1	1	0	0	44	61
**Kosovo**	0	0	0	0	0	0	0	0	0	0	0
**Latvia**	1	1	1	0	0	1	0	0	0	1	2
**Lithuania**	1	2	2	2	0	0	0	0	0	2	4
**Luxembourg**	2	3	3	0	1	2	0	0	0	3	6
**Malta**	0	0	0	0	0	0	0	0	0	0	0
**Montenegro**	0	0	0	0	0	0	0	0	0	0	0
**Netherlands**	8	10	9	0	8	1	0	0	1	9	19
**North Macedonia**	2	0	0	0	0	0	0	0	0	3	3
**Norway**	3	5	4	0	3	1	0	0	1	5	10
**Poland**	1	2	2	1	0	0	1	0	0	2	4
**Portugal**	3	3	3	1	0	2	0	0	0	5	8
**Romania**	1	0	0	0	0	0	0	0	0	7	7
**Serbia**	2	1	1	0	1	0	0	0	0	9	10
**Slovakia**	0	0	0	0	0	0	0	0	0	0	0
**Slovenia**	5	7	6	2	1	3	0	0	1	5	12
**Spain**	10	16	10	0	0	8	2	0	6	19	35
**Sweden**	7	16	16	0	10	6	0	0	0	21	37
**Türkiye**	17	12	10	0	5	4	0	1	2	59	71
**UK**	20	39	30	1	15	13	0	1	9	51	90
**All countries**	156	211	182	21	81	68	7	5	29	337	548

Data are n. I=susceptible, increased exposure. R=resistant. S=susceptible.

Epidemiological characteristics are presented in table 1. Of the 548 *E coli* isolates, 429 (78·3%) originated from clinical samples, 111 (20·3%) from screening samples, and eight (1·5%) from unknown sample types. Urine was the most common sample type in the carbapenem-R/I and carbapenem-S groups. Furthermore, a higher proportion of carbapenem-R/I isolates was recorded as hospital-acquired compared with the proportion of carbapenem-S isolates. The proportion of isolates associated with previous hospital admission (including direct transfer from another hospital) in the past 6 months was also higher among the carbapenem-R/I group than among the carbapenem-S group. Travel to another country within 6 months was more frequently reported in the carbapenem-R/I group than in the carbapenem-S group.

**Figure 1:**
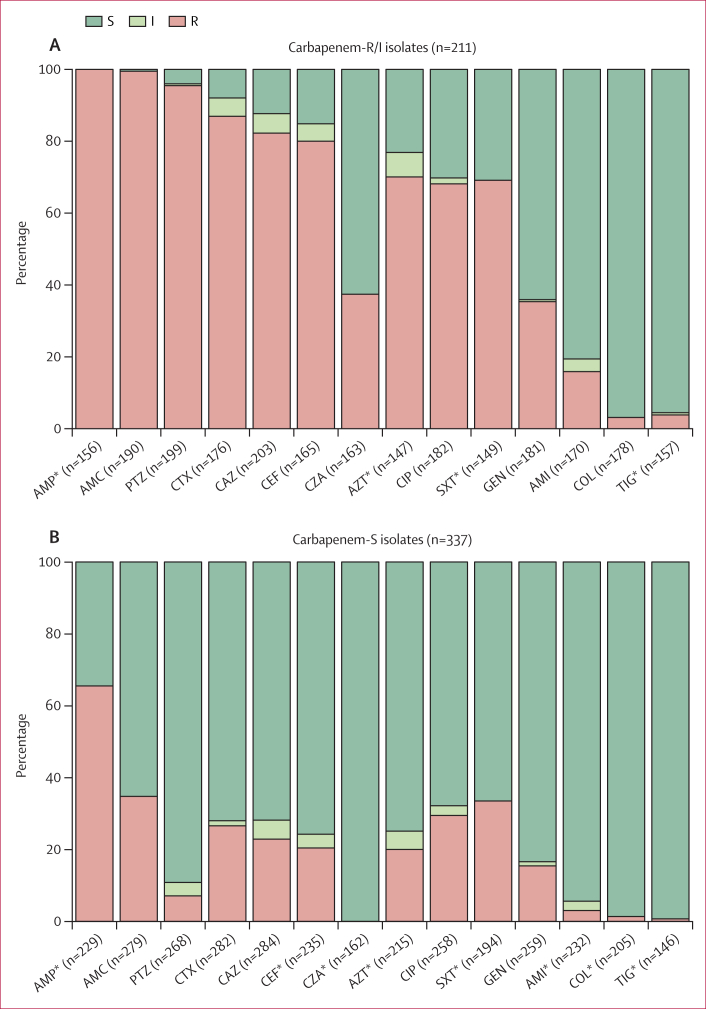
Phenotypic susceptibility for selected antimicrobial agents for 211 carbapenem-R/I (A) and 337 carbapenem-S (B) *Escherichia coli* isolates from the carbapenem-resistant and/or colistin-resistant Enterobacterales survey The numbers of tested isolates are shown in parentheses for each antimicrobial agent. Antimicrobial agents with antimicrobial susceptibility testing results available for less than 75% of isolates are marked with an asterisk. Susceptibility interpretation shown is according to reported antimicrobial susceptibility testing, performed and interpreted by the National Reference Laboratories, according to the European Committee on Antimicrobial Susceptibility Testing (EUCAST) clinical breakpoints table version 9.0, 2019. The number of isolates with antimicrobial susceptibility testing results varies between antimicrobial agents. Note that for colistin, breakpoints are in brackets since the EUCAST breakpoint table version 12.0, 2022. For isolates categorised as colistin-S in the graphs, a combination with another active agent or measure would be required. For aminoglycosides, breakpoints for monotherapy are restricted to infections originating from the urinary tract since EUCAST breakpoint table version 10.0, 2020. AMC=amoxicillin−clavulanic acid. AMI=amikacin. AMP=ampicillin. AZT=aztreonam. CAZ=ceftazidime. CEF=cefepime. CIP=ciprofloxacin. COL=colistin. CTX=cefotaxime. CZA=ceftazidime−avibactam. GEN=gentamicin. I=susceptible, increased exposure. PTZ=piperacillin−tazobactam. R=resistant. S=susceptible. SXT=trimethoprim−sulfamethoxazole. TIG=tigecycline.

More than 75% of carbapenem-R/I *E coli* isolates were tested for susceptibility to amoxicillin–clavulanic acid, piperacillin–tazobactam, cefotaxime, ceftazidime, cefepime, ceftazidime–avibactam, ciprofloxacin, gentamicin, amikacin, and colistin (appendix 1 p 6). High levels of resistance were reported for ciprofloxacin and trimethoprim–sulfamethoxazole for carbapenem-R/I isolates (figure 1, appendix 1 p 2). The two agents with the highest proportion of susceptible isolates were colistin and tigecycline. All reported ceftazidime–avibactam resistance could be explained by the presence of metallo-β-lactamases.

**Figure 2:**
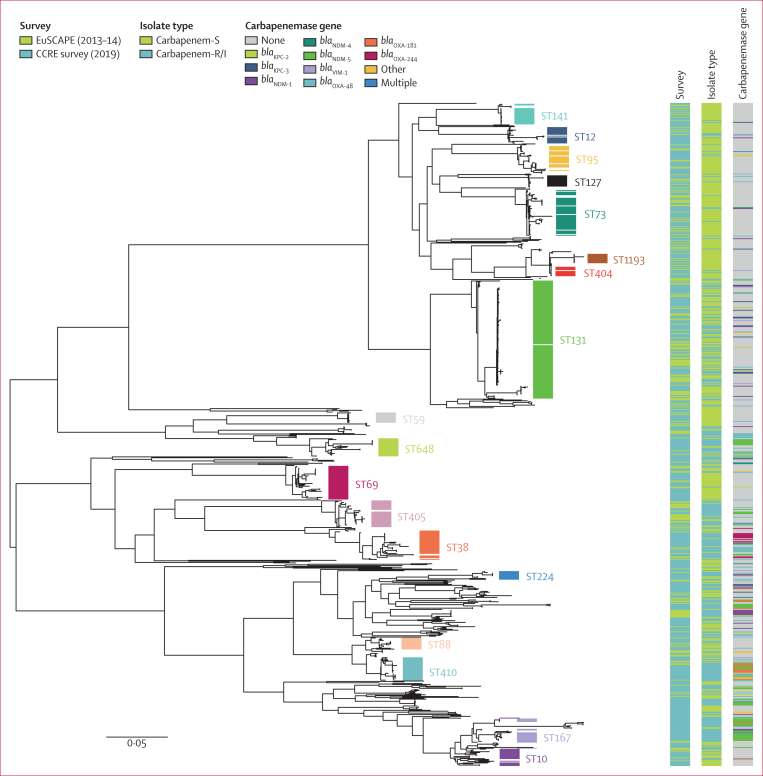
Phylogenetic tree of *Escherichia coli* isolates from the carbapenem-resistant and/or colistin-resistant Enterobacterales survey (n=548) and the 2013–14 European Survey of Carbapenemase-Producing Enterobacteriaceae (n=221) with major STs highlighted on the tree and metadata columns showing the survey, the isolate type (carbapenem-R/I or carbapenem-S), and the carbapenemase gene The scale bar represents the number of single nucleotide polymorphisms per variable site. An interactive version of the tree with additional metadata and genotyping data is available at Microreact. The “Other” category of carbapenemase genes includes *bla*_NDM−7_, *bla*_OXA−162_, *bla*_OXA−204_, *bla*_OXA−232_, *bla*_OXA−427_, and *bla*_OXA−1213_. The “Multiple” category includes the gene combinations *bla*_NDM−1_/*bla*_VIM−86_, *bla*_NDM−1_/*bla*_OXA−232_, *bla*_NDM−5_/*bla*_OXA−181_, and *bla*_NDM−5_/*bla*_OXA−244_. I=susceptible, increased exposure. R=resistant. S=susceptible. ST=sequence type.

Overall, 146 STs were detected among the 548 *E coli* isolates from the CCRE survey, including 12 novel STs. The high diversity of STs is reflected in a phylogenetic tree generated from an alignment of 3310 core genes (length 3 292 772 bp), which also incorporates 221 *E coli* genomes from EuSCAPE (2013–14) that are reported for the first time (figure 2). The 211 carbapenem-R/I *E coli* isolates from the CCRE survey belonged to 74 STs, of which the most common were ST131 (27 [12·8%] of 211), ST410 (20 [9·5%]), ST38 (19 [9·0%]), ST167 (16 [7·6%]), and ST648 (14 [6·6%]). These five STs were found in 13 (ST131), ten (ST410), 13 (ST38), nine (ST167), and seven (ST648) countries (appendix 1 p 7). Although ST131 accounted for the highest number of carbapenem-R/I isolates overall, the proportion of carbapenem-R/I isolates in ST131 (27 [32·5%] of 83) was lower than that in ST410 (20 [90·9%] of 22), ST38 (19 [79·2%] of 24), ST167 (16 [100%] of 16), and ST648 (14 [73·7%] of 19). Among 99 carbapenem-R/I isolates from EuSCAPE (2013–14), ST131 was also the most prevalent ST, accounting for 26 (26%) of 99 isolates, whereas ST410 (four [4·0%]), ST38 (seven [7·1%]), ST167 (two [2·0%]), and ST648 (one [1·0%]) were less common.

The 337 carbapenem-S *E coli* isolates from the CCRE survey belonged to 101 STs. The most common were ST131 (56 [16·6%]), ST69 (30 [8·9%]), ST73 (29 [8·6%]), ST95 (16 [4·7%]), and ST141 (13 [3·9%]). ST131 was also the most frequent ST among carbapenem-S isolates from EuSCAPE, accounting for 27 (22·1%) of 122 isolates.

To further investigate the population structure, we explored whether and how SNP distance patterns varied at different geographical scales (appendix 1 p 8). SNP distances among the 493 isolates included in this analysis ranged from 0 to 93 928 SNPs, reflecting the high diversity among *E coli* isolates. We found that some isolates were highly unrelated to any other isolate in the dataset, with 45 of 493 isolates possessing greater than or equal to 10 000 SNPs to their nearest neighbour. At the other end of the scale, we found that 23 (4·7%) of the 493 isolates had a nearest neighbour in the dataset with less than or equal to 17 SNPs (appendix 1 p 3), a threshold defined by Ludden and colleagues^25^ for predicting nosocomial transmission and which we used here as a proxy for recent transmission. The lowest nearest-neighbour SNP differences were restricted to isolates from the same hospital or country. Five pairs of same-hospital isolates had SNP differences of zero, one, five, seven, and nine, and a further two pairs of same-country isolates (from different hospitals) had SNP differences of zero and one. By contrast, the lowest nearest-neighbour SNP difference for isolates from different countries was 13.

**Figure 3:**
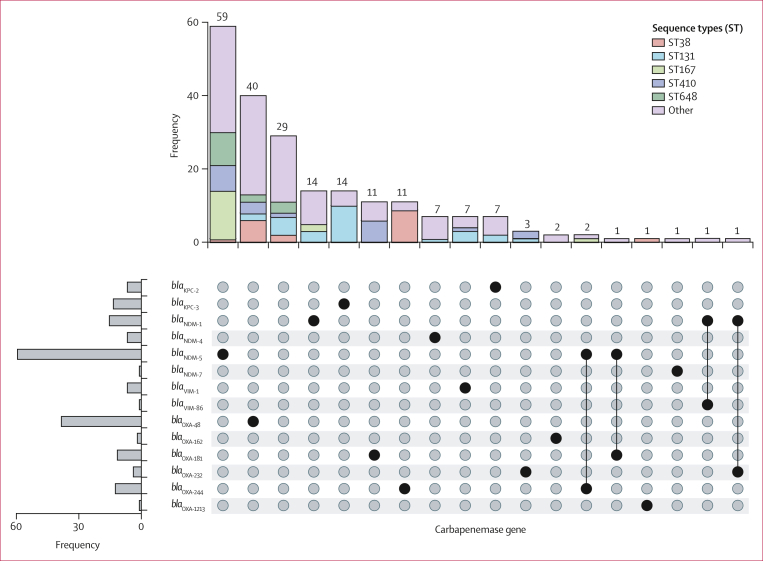
Frequency of major STs and carbapenemase gene variants (or novel related variants) among carbapenem-R/I *Escherichia coli* isolates from the carbapenem-resistant and/or colistin-resistant Enterobacterales survey (n=211) Frequencies of combinations are shown in the upper bar plot, whereas frequencies of individual variants are shown on the left. Twelve of the 14 variants were known carbapenemase genes, whereas two (bla_VIM−86_ and bla_OXA−1213_) were novel variants assigned by GenBank with high similarity to known carbapenemases. ST=sequence type.

One or more carbapenemase genes (or novel related variants) were detected in 182 (86·3%) of 211 carbapenem-R/I *E coli* isolates from the CCRE survey, representing a pronounced increase from EuSCAPE (2013–14) in which 36 (36·4%) of 99 carbapenem-R/I isolates carried a carbapenemase gene. 12 known carbapenemase variants were found among the *E coli* isolates from the CCRE survey, along with two novel variants (*bla*_VIM−86_ and *bla*_OXA−1213_) with high similarity to known carbapenemase genes (figure 3). *bla*_NDM−5_ and *bla*_OXA−48_ dominated, with *bla*_NDM−5_ found in 62 (34·1%) and *bla*_OXA−48_ in 40 (22·0%) of the 182 carbapenemase-positive carbapenem-R/I isolates. Although *bla*_OXA−48_ was also commonly detected among carbapenem-R/I isolates from EuSCAPE (present in 16 of 99 isolates from 2013 to 2014), *bla*_NDM−5_ was found in only two (2·0%) isolates from EuSCAPE.

Carbapenem-R/I isolates were distributed widely across the *E coli* phylogeny and interspersed among carbapenem-S isolates, suggesting numerous independent emergences of carbapenem resistance (figure 2). Among *E coli* isolates from the CCRE survey, individual carbapenemase genes were widely distributed across STs, with *bla*_OXA−48_ being found in 28 and *bla*_NDM−5_ in 17 STs. Some high-risk STs, such as ST131 (22 isolates), carried diverse carbapenemase gene variants: *bla*_KPC−3_, *bla*_NDM−1_, *bla*_VIM−1_, *bla*_KPC−2_, *bla*_OXA−48_, *bla*_OXA−232_, or *bla*_NDM−4_. Of note, among 53 ST131 isolates from EuSCAPE (including 26 carbapenem-R/I isolates), only two carried a carbapenemase gene (*bla*_KPC−2_, *bla*_NDM−1_). We also found associations of carbapenemase genes with particular STs among *E coli* isolates from the CCRE survey (figure 3). For example, five STs, that is, ST167, ST648, ST361, ST410, and ST405, accounted for 43 (69·4%) of the 62 isolates with *bla*_NDM−5_.

Individual carbapenemase genes were geographically widespread, with *bla*_NDM−5_ found among *E coli* isolates from the CCRE survey from 45 hospitals in 15 countries and *bla*_OXA−48_ among isolates from 32 hospitals in 13 countries. We also found varying proportions of different carbapenemase genes among carbapenem-R/I *E coli* isolates by country (table 2, appendix 1 p 9), although this analysis was restricted by the small number of isolates from many countries.

To identify putative plasmid vectors, we examined associations between carbapenemase genes and plasmid replicon types identified in *E coli* isolates from the CCRE survey (appendix 1 p 4), although long-read sequencing would be required for confirmation. We found that 25 (59·5%) of the 42 *E coli* isolates with *bla*_OXA−48_, from 22 STs, carried an IncL replicon, compared with three (0·6%) of the 506 isolates without *bla*_OXA−48_. Multiple replicons were associated with *bla*_NDM−5_-positive *E coli* isolates, including IncFII, IncFIA, IncX3, Col(BS512), and Col(pHAD28).

We also found that carbapenem-R/I isolates carried a higher number of other resistance determinants than carbapenem-S isolates (appendix 1 p 10). In particular, extended-spectrum β-lactamase genes were carried by 131 (62·1%) and acquired AmpC cephalosporinase genes by 28 (13·3%) of the 211 carbapenem-R/I *E coli* isolates. By contrast, among the 337 carbapenem-S isolates, 80 (23·7%) carried extended-spectrum β-lactamase genes and 12 (3·6%) carried acquired AmpC cephalosporinase genes. One carbapenem-R/I and one carbapenem-S isolate carried *mcr*-1, but no colistin minimum inhibitory concentrations were available for these. Two carbapenem-R/I isolates carried the variant *mcr*-9, both with a colistin minimum inhibitory concentration of 2 mg/L.

The increase in the number of isolates carrying *bla*_NDM−5_ between 2013 and 2014 (EuSCAPE) and 2019 (CCRE survey) led us to further investigate the population dynamics of high-risk lineages harbouring this carbapenemase gene. We performed separate phylogenetic analyses of isolates belonging to ST167, ST361, ST405, ST410, and ST648, which had the highest number of *bla*_NDM−5_-carrying isolates in the CCRE survey, incorporating genomes from both surveys and additional publicly available genomes. The publicly available genomes also included isolates from our rapid follow-up investigation of *bla*_NDM−5_-carrying *E coli* initiated from early analysis of these findings.^20^ For each ST, there was a wide distribution of represented countries (≥40). Genomes with *bla*_NDM−5_ dated from 2013 onwards across all five STs, 2 years after the first description of this gene variant.

**Figure 4:**
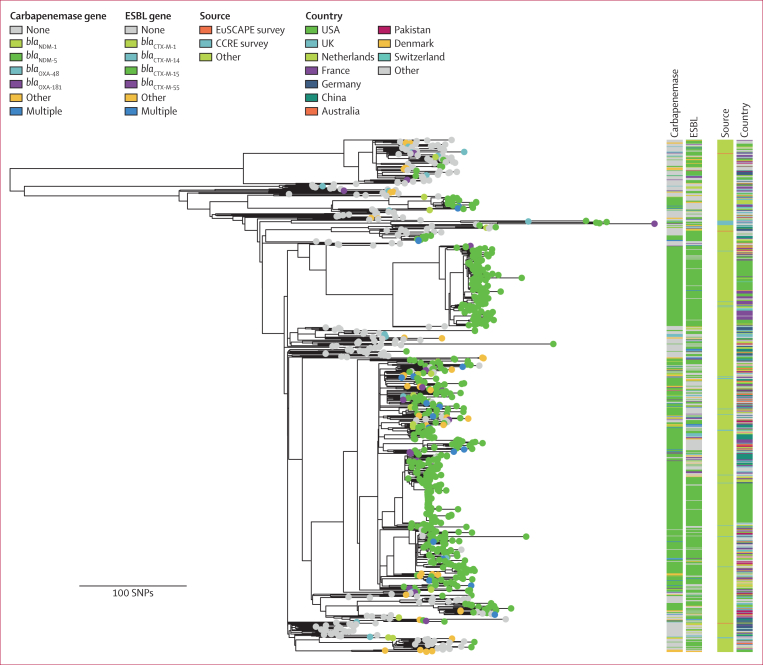
Phylogenetic tree of 899 *Escherichia coli* isolates belonging to ST167 (and other closely related STs) from the carbapenem-resistant and/or colistin-resistant Enterobacterales survey (n=26), 2013–14 European Survey of Carbapenemase-Producing Enterobacteriaceae (n=3), and additional public databases (n=870) Isolate tips are coloured according to the carbapenemase gene variant, and metadata columns show the carbapenemase variant, extended-spectrum β-lactamase variant, data source, and country of origin. The scale bar represents the number of single nucleotide polymorphism (SNPs). An interactive version of the tree with additional metadata and genotyping data is available at Microreact.

Phylogenetic analyses showed that isolates carrying *bla*_NDM−5_ typically belonged to large clonal expansions associated with national and international spread (figure 4, appendix 1 p 11). For ST410 isolates, we also observed substantial clonal spread of an internationally dispersed clade with *bla*_OXA−181_ (appendix 1 p 11). In each ST, *bla*_NDM−5_ was found in multiple clades, suggesting that this gene has been acquired independently multiple times. Notably, we found that isolates from individual countries, including those from European countries, were widely distributed across each of the phylogenies, typically appearing as singletons or within small clusters of isolates from the same country. Clonal expansions of *bla*_NDM−5_-carrying isolates were also typically associated with genes conferring resistance to other antibiotic classes (see Microreact URLs from figure 4 and appendix 1 p 11).

## Discussion

We report results from the CCRE survey for 548 *E coli* isolates from 32 European countries in 2019. Although, as in EuSCAPE (2013–14), carbapenem-R/I *E coli* isolates were outnumbered by carbapenem-R/I *K pneumoniae*,^4^ we detected various signals of a worsening epidemiological situation for carbapenem-R/I *E coli*. Of particular concern, we found that carbapenem-R/I *E coli* isolates mainly belonged to high-risk STs associated with multidrug resistance and confirmed substantial clonal spread of these high-risk STs, driving national and international spread of carbapenemase genes. Only minimal local transmission was detected within the studied European countries; however, the ability to detect transmission might be restricted by the design of the CCRE survey, the choice of the SNP threshold, and incomplete surveillance in several countries.

Although the small number of isolates, different participating hospitals in the surveys, and additional inclusion of screening isolates in the CCRE survey did not allow a more detailed comparison of *E coli* isolates from the two surveys, we detected two major differences. The first was the increase in the proportion of carbapenem-R/I isolates with a carbapenemase gene. Because of the wide reservoir of *E coli* strains, a rising prevalence of highly mobilisable carbapenemase genes represents a formidable challenge to controlling the spread of carbapenem resistance. Fortunately, phenotypic colistin resistance and carriage of *mcr* genes have remained low among carbapenem-R/I isolates.

The second key difference between the two surveys was the increase in the number of isolates carrying *bla*_NDM−5_ among carbapenem-R/I *E coli* isolates. Our rapid follow-up study using national routine WGS data initiated after a preliminary analysis of CCRE survey data confirmed the increase in *bla*_NDM−5_-carrying *E coli* across European countries between 2012 and 2022.^20^ Since the first identification of *bla*_NDM−5_ in *E coli* in 2011, an increasing number of studies have reported its occurrence in different regions worldwide.^26^^,^^27^ Notably, *bla*_NDM−5_ has enhanced carbapenemase activity compared with *bla*_NDM−1_.^28^ Analysis of associated replicon types suggested that several plasmid types might be serving as vectors, consistent with other reports.^28^

The high-risk STs identified in this study have also been shown to dominate other carbapenemase-producing *E coli* collections.^29^ Studies have shown that acquisition of carbapenem resistance frequently occurs in already multidrug-resistant lineages, as exemplified in ST410.^30^ Concentration of carbapenemase-producing isolates in high-risk STs could be explained by different scenarios that might co-occur and have had varying significance in different STs at the time of the CCRE survey. For example, in the dominant pathogenic ST131 lineage of *E coli*, we observed numerous independent acquisitions of different carbapenemase genes. These might represent repeated emergence of carbapenem resistance among locally circulating lineages.

Conversely, *E coli* isolates belonging to ST167, ST361, ST405, ST410, and ST648 were predominantly carbapenem-R/I, and phylogenetic analyses showed substantial clonal spread of globally distributed *bla*_NDM−5_-carrying lineages from these STs. The large and densely sampled lineages of *bla*_NDM−5_-carrying isolates are a major cause for concern as they indicate a pathogen that is disseminating rapidly, having successfully adapted to the carriage of the associated plasmid(s). Carbapenem-R/I *E coli* isolates from the studied European countries largely occurred as singletons or in small clusters in the global phylogenies of these five STs, demonstrating numerous imports into these European countries with minimal onward local transmission. This finding was supported by our SNP analysis of *E coli* isolates from the CCRE survey, in which we found only five geographically linked pairs. Surveillance based on data within the past five years of *E coli* carrying *bla*_NDM−5_ and *bla*_OXA−244_ also suggested little local spread in either hospital or community settings within Europe.^20^^,^^31^

This study has several limitations. The study used a surveillance survey involving voluntary participation of sentinel hospitals, with the primary aim of collecting isolates for molecular typing with a harmonised approach and wide geographical coverage. There was an uneven distribution of *E coli* isolates from different European countries in the CCRE survey, and an uneven distribution of submitted isolates by sample type, both between countries and between the carbapenem-R/I and carbapenem-S groups. Inclusion was based on EUCAST clinical breakpoints and not on the recommended screening breakpoints, which might have resulted in underdetection of carbapenemase genes frequently associated with low carbapenem minimum inhibitory concentration, such as *bla*_OXA−48_−like. The CCRE survey was also not designed for comprehensive detection of national trends, regional outbreaks, and onward transmissions because of the restricted number of included hospitals and isolates per hospital.

In conclusion, we report the emergence of carbapenemase-producing *E coli* in European countries driven by the clonal global spread of several high-risk STs. A rise in carbapenem resistance similar to the rapid pace at which high-risk cephalosporin-resistant and fluoroquinolone-resistant lineages of *E coli* spread would pose an unprecedented threat to patients with *E coli* infections in Europe and worldwide and demands urgent global public health efforts to improve early detection, WGS-based surveillance, infection prevention and control, and antimicrobial stewardship.

## Data sharing

Whole-genome sequencing data for this study were deposited in the European Nucleotide Archive under accession numbers PRJEB39943 and PRJEB51224 (CCRE survey isolates) and PRJEB10018 (EuSCAPE isolates). Interactive dashboards containing a phylogeny and metadata are available at Microreact with further URLs for individual high-risk STs provided in appendix 1 (p 11).

## Declaration of interests

IF, SB, and AB report funding from ECDC through the framework contract ECDC/2017/021. EM reports being part of the EUCAST Executive and General Committees as Head of the EUCAST development laboratory and Co-chair of the CLSI-EUCAST joint working group. KTW reports being a National Microbiology Focal Point (2014–21) and a Board Member (2021–24) for ECDC and a Member of the Steering Group on Health Promotion, Disease Prevention, and Management of Non-Communicable Diseases (2022–23) and of the Expert Group on Public Health (2023–24) of the European Commission (Directorate-General for Health and Food Safety [DG SANTE]). DMA reports grants to the University of Oxford from the National Institute for Health and Care Research, UK (grant number NIHR133307) and core funding to the Centre for Genomic Pathogen Surveillance from the Wellcome Sanger Institute. All other authors declare no competing interests.
