# Supplementary material for: Emergence of carbapenemase-producing Escherichia coli in acute care hospitals in 32 European countries (the CCRE survey): a prospective, multicentre, cross-sectional, epidemiological, microbiological, and genomic surveillance study
Source: Lancet Microbe. 2026 Jun;7(6):None. doi: 10.1016/j.lanmic.2025.101321 (PMC13259978; doi:10.1016/j.lanmic.2025.101321)
Supplement: Supplementary appendix 1 [file mmc1.pdf]

THE LANCET
Microbe

Supplementary appendix 1

This appendix formed part of the original submission and has been peer reviewed. We post it as supplied by the authors.

Supplement to: David S, Kohlenberg A, Yeats C, et al. Emergence of carbapenemase-producing *Escherichia coli* in acute care hospitals in 32 European countries (the CCRE survey): a prospective, multicentre, cross-sectional, epidemiological, microbiological, and genomic surveillance study. *Lancet Microbe* 2026. https://doi.org/10.1016/j.lanmic.2025.101321

Table of Contents

Table A1 Susceptibility to antimicrobials other than carbapenems in *Escherichia coli* isolates from the carbapenem- and/or colistin-resistant Enterobacterales (CCRE) survey 2

Table A2 *Escherichia coli* isolates from the carbapenem- and/or colistin-resistant Enterobacterales (CCRE) survey with a nearest neighbour in the data set with ≤17 single nucleotide polymorphisms .. 3

Table A3 Percentage of *Escherichia coli* genomes from the carbapenem- and/or colistin-resistant Enterobacterales (CCRE) survey with and without particular carbapenemase gene variants carrying individual plasmid replicons 4

Figure A1 Flowchart of *Klebsiella pneumoniae* SC* and *Escherichia coli* isolates in the carbapenem- and/or colistin-resistant Enterobacterales (CCRE) survey 5

Figure A2 Percentage of 211 carbapenem-R/I and 337 carbapenem-S *Escherichia coli* isolates from the carbapenem- and/or colistin-resistant Enterobacterales (CCRE) survey with phenotypic antimicrobial susceptibility testing results 6

Figure A3 Country distribution of major sequence types among 211 carbapenem-R/I *Escherichia coli* isolates from the carbapenem- and/or colistin-resistant Enterobacterales (CCRE) survey 7

Figure A4 SNP differences between *Escherichia coli* isolates from the carbapenem- and/or colistin-resistant Enterobacterales (CCRE) survey and their nearest neighbours from the same hospital (A-C), a different hospital in the same country (D-F) and a different country (G-I) 8

Figure A5 Distribution of carbapenemase genes detected in 211 carbapenem-R/I *Escherichia coli* isolates from the carbapenem- and/or colistin-resistant Enterobacterales (CCRE) survey, by country 9

Figure A6 Combinations of resistance genes and mutations among (A) 211 carbapenem-R/I and (B) 337 carbapenem-S *Escherichia coli* isolates from the carbapenem- and/or colistin-resistant Enterobacterales (CCRE) survey 10

Figure A7 Phylogenetic trees of *Escherichia coli* isolates belonging to (A) ST361 (n=374), (B) ST405 (n=974), (C) ST410 (n=1182) and (D) ST648 (n=789) 11

Table A1 Susceptibility to antimicrobials other than carbapenems in *Escherichia coli* isolates from the carbapenem- and/or colistin-resistant Enterobacterales (CCRE) survey

	Carbapenem-R/I *E. coli*				Carbapenem-R/I *E. coli* carrying carbapenemase genes				Carbapenem-S *E. coli*			
	**n tested**	**R n (%, 95%CI)**	**I n (%, 95%CI)**	**S n (%, 95%CI)**	**n tested**	**R n (%, 95%CI)**	**I n (%, 95%CI)**	**S n (%, 95%CI)**	**n tested**	**R n (%, 95%CI)**	**I n (%, 95%CI)**	**S n (%, 95%CI)**
Ampicillin	156	156 (100·0, 100·0-100·0)	NA	0	130	130 (100·0, 100·0-100·0)	NA	0	229	150 (65·5, 52·7-76·4)	NA	79 (34·5, 23·6-47·3)
Amoxicillin-clavulanic acid	190	189 (99·5, 96·5-99·9)	NA	1 (0·5, 0·1-3·5)	161	160 (99·4, 95·7-99·9)	NA	1 (0·6, 0·1-4·3)	279	97 (34·8, 27·7-42·6)	NA	182 (65·2, 57·4-72·3)
Piperacillin-tazobactam	199	190 (95·5, 91·1-97·7)	1 (0·5, 0·1-3·3)	8 (4·0, 1·8-8·9)	170	168 (98·8, 95·0-99·7)	0	2 (1·2, 0·3-5·0)	268	19 (7·1, 3·7-13·3)	10 (3·7, 2·0-6·9)	239 (89·2, 83·0-93·3)
Cefotaxime	176	153 (86·9, 74·0-94·0)	9 (5·1, 1·5-16·4)	14 (8·0, 4·3-14·2)	147	127 (86·4, 73·6-93·5)	8 (5·4, 1·6-17·2)	12 (8·2, 4·8-13·5)	282	75 (26·6, 18·3-36·9)	4 (1·4, 0·6-3·4)	203 (72·0, 61·9-80·2)
Ceftazidime	203	167 (82·3, 71·4-89·6)	11 (5·4, 3·2-9·1)	25 (12·3, 6·3-22·6)	174	143 (82·2, 71·6-89·4)	8 (4·6, 2·8-7·6)	23 (13·2, 6·9-23·8)	284	65 (22·9, 15·4-32·6)	15 (5·3, 2·7-10·0)	204 (71·8, 62·6-79·6)
Cefepime	165	132 (80·0, 67·2-88·6)	8 (4·8, 2·3-9·8)	25 (15·2, 7·9-27·2)	137	108 (78·8, 63·0-89·1)	7 (5·1, 2·5-10·1)	22 (16·1, 7·7-30·6)	235	48 (20·4, 13·2-30·2)	9 (3·8, 1·8-8·1)	178 (75·7, 65·0-84·0)
Ceftazidime-avibactam	163	61 (37·4, 26·9-49·3)	NA	102 (62·6, 50·7-73·1)	138	61 (44·2, 31·8-57·4)	NA	77 (55·8, 42·6-68·2)	162	0	NA	162 (100·0, 100·0-100·0)
Aztreonam	147	103 (70·1, 54·2-82·2)	10 (6·8, 3·5-12·9)	34 (23·1, 12·3-39·3)	120	82 (68·3, 52·4-80·9)	7 (5·8, 2·5-12·8)	31 (25·8, 14·3-42·2)	215	43 (20·0, 12·1-31·2)	11 (5·1, 2·9-9·0)	161 (74·9, 63·0-83·9)
Ciprofloxacin	182	124 (68·1, 61·5-74·1)	3 (1·6, 0·7-3·8)	55 (30·2, 23·8-37·5)	157	103 (65·6, 56·9-73·4)	3 (1·9, 0·8-4·4)	51 (32·5, 24·2-42·1)	258	76 (29·5, 20·8-40·0)	7 (2·7, 1·4-5·1)	175 (67·8, 57·4-76·8)
Trimethoprim-sulfamethoxazole	149	103 (69·1, 58·3-78·2)	0	46 (30·9, 21·8-41·7)	133	93 (69·9, 57·6-79·9)	0	40 (30·1, 20·1-42·4)	194	65 (33·5, 27·7-39·9)	0	129 (66·5, 60·1-72·3)
Gentamicin	181	64 (35·4, 29·1-42·2)	1 (0·6, 0·1-3·7)	116 (64·1, 57·4-70·3)	156	54 (34·6, 27·2-42·8)	1 (0·6, 0·1-4·1)	101 (64·7, 56·7-72·1)	259	40 (15·4, 10·5-22·1)	3 (1·2, 0·4-3·3)	216 (83·4, 77·3-88·1)
Tobramycin	119	47 (39·5, 27·9-52·4)	4 (3·4, 1·2-9·0)	68 (57·1, 43·5-69·8)	106	44 (41·5, 30·0-54·0)	4 (3·8, 1·4-9·6)	58 (54·7, 41·6-67·2)	155	33 (21·3, 15·5-28·5)	4 (2·6, 1·0-6·4)	118 (76·1, 69·4-81·8)
Amikacin	170	27 (15·9, 9·6-25·2)	6 (3·5, 1·3-9·3)	137 (80·6, 69·2-88·5)	146	26 (17·8, 10·8-27·9)	6 (4·1, 1·5-10·5)	114 (78·1, 65·8-86·9)	232	7 (3·0, 1·3-6·9)	6 (2·6, 0·9-7·1)	219 (94·4, 88·1-97·5)
Colistin	178	6 (3·4, 1·3-8·8)	NA	172 (96·6, 91·2-98·7)	158	4 (2·5, 0·8-7·7)	NA	154 (97·5, 92·3-99·2)	205	3 (1·5, 0·5-4·6)	NA	202 (98·5, 95·4-99·5)
Tigecycline	156	6 (3·8, 1·8-8·0)	NA	150 (96·2, 92·0-98·2)	135	5 (3·7, 1·5-9·1)	NA	130 (96·3, 90·9-98·5)	146	1 (0·7, 0·1-4·6)	NA	145 (99·3, 95·4-99·9)
Fosfomycin	97	9 (9·3, 5·5-15·1)	NA	88 90·7, 84·9-94·5)	85	6 (7·1, 4·2-11·7)	NA	79 (92·9, 88·3-95·8)	65	5 (7·7, 2·8-19·6)	NA	60 (92·3, 80·4-97·2)

R, resistant; I, susceptible, increased exposure; S, susceptible; NA, not available, 95%CI = 95% confidence interval.
The table shows the number of tested isolates and proportion of tested isolates that were reported resistant (R), susceptible, increased exposure (I) and susceptible (S) with 95% confidence intervals, to each antimicrobial agent for 211 carbapenem-R/I E. coli isolates, 182 carbapenem-R/I E. coli isolates carrying carbapenemase genes and 337 carbapenem-S E. coli isolates. Results are from antimicrobial susceptibility tests (AST), performed and interpreted by the NRLs, according to EUCAST clinical breakpoints table v9.0, 2019. The number of isolates with AST results varies between antimicrobial agents. For some antimicrobials breakpoints have been changed in subsequent years. For piperacillin-tazobactam, isolates categorised as I in 2019, would be categorised as R from v11.0, 2021. For aminoglycosides, breakpoints for monotherapy are limited to infections originating from the urinary tract, and isolates categorized as I for aminoglycosides in 2019, would be categorised as R since EUCAST breakpoint table v10.0, 2020. The proportion of isolates that were tested for tobramycin and fosfomycin was <60%, for this reason these results are considered uncertain, and the results were not included in Figure 1. Note that for colistin, breakpoints are in brackets since the EUCAST breakpoint table v12.0, 2022. For isolates categorised as colistin S in the graphs, combination with another active agent or measure would be required.

Table A2 *Escherichia coli* isolates from the carbapenem- and/or colistin-resistant Enterobacterales (CCRE) survey with a nearest neighbour in the data set with ≤17 single nucleotide polymorphisms

Isolate ID	Isolate type	Country	Hospital code	ST	Carbapene-mase gene	Nearest-neighbour SNP distance
TR03_S05	Carbapenem-S	Türkiye	TR03	10	-	0 SNPs with TR03_S08 (same hospital)
TR03_S08	Carbapenem-S	Türkiye	TR03	10	-	0 SNPs with TR03_S05 (same hospital
IT45_R01	Carbapenem-R/I	Italy	IT45	131	bla_{KPC-3}	1 SNP with IT45_R02 (same hospital)
IT45_R02	Carbapenem-R/I	Italy	IT45	131	bla_{KPC-3}	1 SNP with IT45_R01 (same hospital)
ES23_R01	Carbapenem-R/I	Spain	ES23	88	-	5 SNPs with ES23_R02 (same hospital)
ES23_R02	Carbapenem-R/I	Spain	ES23	88	-	5 SNPs with ES23_R01 (same hospital)
UK12_R05	Carbapenem-R/I	United Kingdom	UK12	1284	bla_{NDM-5}	7 SNPs with UK12_R06 (same hospital)
UK12_R06	Carbapenem-R/I	United Kingdom	UK12	1284	bla_{NDM-5}	7 SNPs with UK12_R05 (same hospital)
DE03_R02	Carbapenem-R/I	Germany	DE03	744	-	9 SNPs with DE03_S02 (same hospital)
DE03_S02	Carbapenem-S	Germany	DE03	744	-	9 SNPs with DE03_R02 (same hospital)
IT22_S09	Carbapenem-S	Italy	IT22	131	-	0 SNPs with IT14_S06 (different hospital)
IT14_S06	Carbapenem-S	Italy	IT14	131	-	0 SNPs with IT22_S09 (different hospital)
ES07_R01	Carbapenem-R/I	Spain	ES07	5968	$bla_{OXA-162}$	1 SNP with ES17_R10 (different hospital)
ES17_R10	Carbapenem-R/I	Spain	ES17	5968	$bla_{OXA-162}$	1 SNP with ES07_R01 (different hospital)
DE18_S02	Carbapenem-S	Germany	DE18	95	-	13 SNPs with SE01_S05 (different country)
SE01_S05	Carbapenem-S	Sweden	SE01	95	-	13 SNPs with DE18_S02 (different country)
LU04_R02	Carbapenem-R/I	Luxembourg	LU04	38	$bla_{OXA-244}$	14 SNPs with LV01_R02 (different country)
LV01_R02	Carbapenem-R/I	Latvia	LV01	38	$bla_{OXA-244}$	14 SNPs with LU04_R02 (different country)
IT38_S09	Carbapenem-S	Italy	IT38	69	-	17 SNPs with UK12_S05 and TR12_S09 (different country)
UK12_S05	Carbapenem-S	United Kingdom	UK12	69	-	17 SNPs with IT38_S09 (different country)
TR12_S09	Carbapenem-S	Türkiye	TR12	69	-	17 SNPs with IT38_S09 (different country)
NL34_R02	Carbapenem-R/I	Netherlands	NL34	167	bla_{NDM-5}	17 SNPs with UK22_R03 (different country)
UK22_R03	Carbapenem-R/I	United Kingdom	UK22	167	bla_{NDM-5}	17 SNPs with NL34_R02 (different country)

ID, identifier; ST, sequence type; S, susceptible; R, resistant; I, susceptible, increased exposure; SNP, single nucleotide polymorphism.

Table A3 Percentage of *Escherichia coli* genomes from the carbapenem- and/or colistin-resistant Enterobacterales (CCRE) survey with and without particular carbapenemase gene variants carrying individual plasmid replicons

	Carbapenemase	KPC-2		KPC-3		NDM-1		NDM-4		NDM-5		VIM-1		OXA-48		OXA-162		OXA-181		OXA-232		OXA-244	
		Yes	No	Yes	No	Yes	No	Yes	No	Yes	No	Yes	No	Yes	No	Yes	No	Yes	No	Yes	No	Yes	No
Plasmid replicon	No. of isolates	7	541	14	534	16	532	7	541	62	486	7	541	42	506	2	546	12	536	4	544	13	535
IncFIB(AP001918)_1__AP001918	331	85.7	60.1	71.4	60.1	50.0	60.7	71.4	60.3	54.8	61.1	71.4	60.3	45.2	61.7	0.0	60.6	66.7	60.3	50.0	60.5	76.9	60.0
IncFII_1__AY458016	209	28.6	38.3	28.6	38.4	25.0	38.5	42.9	38.1	66.1	34.6	42.9	38.1	21.4	39.5	0.0	38.3	8.3	38.8	50.0	38.1	84.6	37.0
IncFIA_1__AP001918	175	14.3	32.2	57.1	31.3	12.5	32.5	14.3	32.2	77.4	26.1	28.6	32.0	28.6	32.2	0.0	32.1	58.3	31.3	75.0	31.6	23.1	32.1
Col156_1__NC_009781	157	28.6	28.7	35.7	28.5	18.8	28.9	0.0	29.0	16.1	30.2	42.9	28.5	28.6	28.7	0.0	28.8	0.0	29.3	25.0	28.7	15.4	29.0
Col(MG828)_1__NC_008486	82	14.3	15.0	14.3	15.0	12.5	15.0	0.0	15.2	22.6	14.0	14.3	15.0	19.0	14.6	0.0	15.0	0.0	15.3	25.0	14.9	0.0	15.3
IncI1-I(Gamma)_1__AP005147	67	0.0	12.4	21.4	12.0	31.3	11.7	57.1	11.6	14.5	11.9	0.0	12.4	19.0	11.7	0.0	12.3	8.3	12.3	25.0	12.1	7.7	12.3
IncFII(pRSB107)_1__AJ851089	63	0.0	11.6	35.7	10.9	6.3	11.7	0.0	11.6	9.7	11.7	0.0	11.6	2.4	12.3	0.0	11.5	0.0	11.8	25.0	11.4	0.0	11.8
Col(BS512)_1__NC_010656	60	0.0	11.1	0.0	11.2	0.0	11.3	0.0	11.1	30.6	8.4	14.3	10.9	14.3	10.7	0.0	11.0	41.7	10.3	50.0	10.7	7.7	11.0
Col(pHAD28)_1__KU674895	52	14.3	9.4	7.1	9.6	12.5	9.4	0.0	9.6	30.6	6.8	0.0	9.6	7.1	9.7	0.0	9.5	16.7	9.3	25.0	9.4	7.7	9.5
IncFII(pSE11)_1__AP009242	40	0.0	7.4	7.1	7.3	25.0	6.8	28.6	7.0	1.6	8.0	28.6	7.0	7.1	7.3	0.0	7.3	0.0	7.5	0.0	7.4	0.0	7.5
IncX3_1__JN247852	40	0.0	7.4	28.6	6.7	12.5	7.1	100.0	6.1	22.6	5.3	14.3	7.2	0.0	7.9	0.0	7.3	75.0	5.8	0.0	7.4	0.0	7.5
IncFII(29)_1__CP003035	39	14.3	7.0	0.0	7.3	12.5	7.0	14.3	7.0	1.6	7.8	14.3	7.0	2.4	7.5	0.0	7.1	0.0	7.3	0.0	7.2	0.0	7.3
IncX1_1__EU370913	32	0.0	5.9	7.1	5.8	12.5	5.6	0.0	5.9	0.0	6.6	0.0	5.9	11.9	5.3	0.0	5.9	0.0	6.0	0.0	5.9	7.7	5.8
ColRNAI_1__DQ298019	31	14.3	5.5	0.0	5.8	0.0	5.8	0.0	5.7	11.3	4.9	28.6	5.4	4.8	5.7	0.0	5.7	0.0	5.8	0.0	5.7	0.0	5.8
IncFII(pCoo)_1__CR942285	31	14.3	5.5	0.0	5.8	6.3	5.6	0.0	5.7	6.5	5.6	0.0	5.7	11.9	5.1	0.0	5.7	16.7	5.4	0.0	5.7	0.0	5.8
IncY_1__K02380	29	14.3	5.2	14.3	5.1	6.3	5.3	0.0	5.4	11.3	4.5	0.0	5.4	7.1	5.1	0.0	5.3	16.7	5.0	50.0	5.0	7.7	5.2
IncL_1__JN626286	28	0.0	5.2	0.0	5.2	0.0	5.3	0.0	5.2	0.0	5.8	0.0	5.2	59.5	0.6	100.0	4.8	0.0	5.2	0.0	5.1	0.0	5.2
p0111_1__AP010962	28	0.0	5.2	0.0	5.2	6.3	5.1	0.0	5.2	8.1	4.7	0.0	5.2	14.3	4.3	0.0	5.1	0.0	5.2	0.0	5.1	0.0	5.2
IncFII(pAMA1167-NDM-5)_1__CP024805	22	0.0	4.1	0.0	4.1	0.0	4.1	0.0	4.1	14.5	2.7	0.0	4.1	14.3	3.2	0.0	4.0	58.3	2.8	0.0	4.0	7.7	3.9
IncX4_2__FN543504	20	0.0	3.7	7.1	3.6	0.0	3.8	0.0	3.7	6.5	3.3	14.3	3.5	2.4	3.8	0.0	3.7	8.3	3.5	0.0	3.7	0.0	3.7
IncB/O/K/Z_1__CU928147	18	0.0	3.3	0.0	3.4	0.0	3.4	14.3	3.1	3.2	3.3	0.0	3.3	2.4	3.4	0.0	3.3	0.0	3.4	0.0	3.3	7.7	3.2
IncFIB(H89-PhagePlasmid)_1__HG530657	18	0.0	3.3	0.0	3.4	0.0	3.4	0.0	3.3	16.1	1.6	0.0	3.3	0.0	3.6	0.0	3.3	0.0	3.4	0.0	3.3	15.4	3.0
IncFII(K)_1__CP000648	17	28.6	2.8	64.3	1.5	12.5	2.8	0.0	3.1	1.6	3.3	0.0	3.1	2.4	3.2	0.0	3.1	0.0	3.2	0.0	3.1	0.0	3.2
IncB/O/K/Z_2__GU256641	17	0.0	3.1	0.0	3.2	6.3	3.0	0.0	3.1	1.6	3.3	0.0	3.1	2.4	3.2	0.0	3.1	0.0	3.2	0.0	3.1	0.0	3.2
ColpVC_1__JX133088	16	0.0	3.0	0.0	3.0	0.0	3.0	28.6	2.6	3.2	2.9	0.0	3.0	7.1	2.6	0.0	2.9	0.0	3.0	0.0	2.9	0.0	3.0
IncFIB(pQil)_1__JN233705	15	42.9	2.2	64.3	1.1	6.3	2.6	0.0	2.8	1.6	2.9	0.0	2.8	0.0	3.0	0.0	2.7	0.0	2.8	0.0	2.8	0.0	2.8
Col440I_1__CP023920	13	0.0	2.4	0.0	2.4	0.0	2.4	0.0	2.4	3.2	2.3	0.0	2.4	9.5	1.8	0.0	2.4	8.3	2.2	0.0	2.4	0.0	2.4
IncI2(Delta)_1__AP002527	13	0.0	2.4	0.0	2.4	6.3	2.3	0.0	2.4	3.2	2.3	0.0	2.4	2.4	2.4	0.0	2.4	8.3	2.2	0.0	2.4	0.0	2.4
IncX4_1__CP002895	13	0.0	2.4	0.0	2.4	0.0	2.4	0.0	2.4	1.6	2.5	0.0	2.4	2.4	2.4	0.0	2.4	8.3	2.2	0.0	2.4	0.0	2.4
IncN_1__AY046276	12	42.9	1.7	0.0	2.2	6.3	2.1	0.0	2.2	0.0	2.5	14.3	2.0	0.0	2.4	0.0	2.2	0.0	2.2	0.0	2.2	0.0	2.2
IncFIC(FII)_1__AP001918	12	28.6	1.8	0.0	2.2	0.0	2.3	0.0	2.2	0.0	2.5	0.0	2.2	2.4	2.2	0.0	2.2	0.0	2.2	0.0	2.2	0.0	2.2
IncC_1__JN157804	12	0.0	2.2	0.0	2.2	56.3	0.6	0.0	2.2	1.6	2.3	14.3	2.0	0.0	2.4	0.0	2.2	0.0	2.2	0.0	2.2	0.0	2.2
Col8282_1__DQ995352	11	0.0	2.0	0.0	2.1	0.0	2.1	0.0	2.0	3.2	1.9	0.0	2.0	4.8	1.8	0.0	2.0	0.0	2.1	0.0	2.0	0.0	2.1
IncFIB(K)_1__JN233704	9	0.0	1.7	7.1	1.5	0.0	1.7	0.0	1.7	1.6	1.6	0.0	1.7	2.4	1.6	0.0	1.6	0.0	1.7	0.0	1.7	0.0	1.7
IncFII(pHN7A8)_1__JN232517	9	0.0	1.7	0.0	1.7	6.3	1.5	0.0	1.7	0.0	1.9	0.0	1.7	4.8	1.4	0.0	1.6	0.0	1.7	0.0	1.7	15.4	1.3
IncFIB(pB171)_1__AB024946	8	0.0	1.5	0.0	1.5	18.8	0.9	0.0	1.5	4.8	1.0	0.0	1.5	0.0	1.6	0.0	1.5	0.0	1.5	25.0	1.3	0.0	1.5
IncB/O/K/Z_4__FN868832	8	0.0	1.5	0.0	1.5	0.0	1.5	14.3	1.3	0.0	1.6	0.0	1.5	0.0	1.6	0.0	1.5	0.0	1.5	0.0	1.5	7.7	1.3
IncHI2_1__BX664015	7	0.0	1.3	0.0	1.3	0.0	1.3	0.0	1.3	0.0	1.4	28.6	0.9	7.1	0.8	0.0	1.3	0.0	1.3	0.0	1.3	0.0	1.3
IncHI2A_1__BX664015	7	0.0	1.3	0.0	1.3	0.0	1.3	0.0	1.3	0.0	1.4	28.6	0.9	7.1	0.8	0.0	1.3	0.0	1.3	0.0	1.3	0.0	1.3
IncR_1__DQ449578	6	14.3	0.9	7.1	0.9	0.0	1.1	0.0	1.1	0.0	1.2	0.0	1.1	2.4	1.0	0.0	1.1	8.3	0.9	0.0	1.1	0.0	1.1
IncHI1A_1__AF250878	5	14.3	0.7	0.0	0.9	0.0	0.9	0.0	0.9	0.0	1.0	0.0	0.9	0.0	1.0	0.0	0.9	0.0	0.9	0.0	0.9	0.0	0.9
IncHI1B(R27)_1_R27_AF250878	5	14.3	0.7	0.0	0.9	0.0	0.9	0.0	0.9	0.0	1.0	0.0	0.9	0.0	1.0	0.0	0.9	0.0	0.9	0.0	0.9	0.0	0.9
Col440II_1__CP023921	5	0.0	0.9	0.0	0.9	6.3	0.8	0.0	0.9	0.0	1.0	0.0	0.9	0.0	1.0	0.0	0.9	0.0	0.9	0.0	0.9	0.0	0.9
IncX1_4__JN935898	5	28.6	0.6	0.0	0.9	0.0	0.9	0.0	0.9	1.6	0.8	14.3	0.7	0.0	1.0	0.0	0.9	0.0	0.9	0.0	0.9	0.0	0.9

Figure A1 Flowchart of *Klebsiella pneumoniae* SC* and *Escherichia coli* isolates in the carbapenem- and/or colistin-resistant Enterobacterales (CCRE) survey

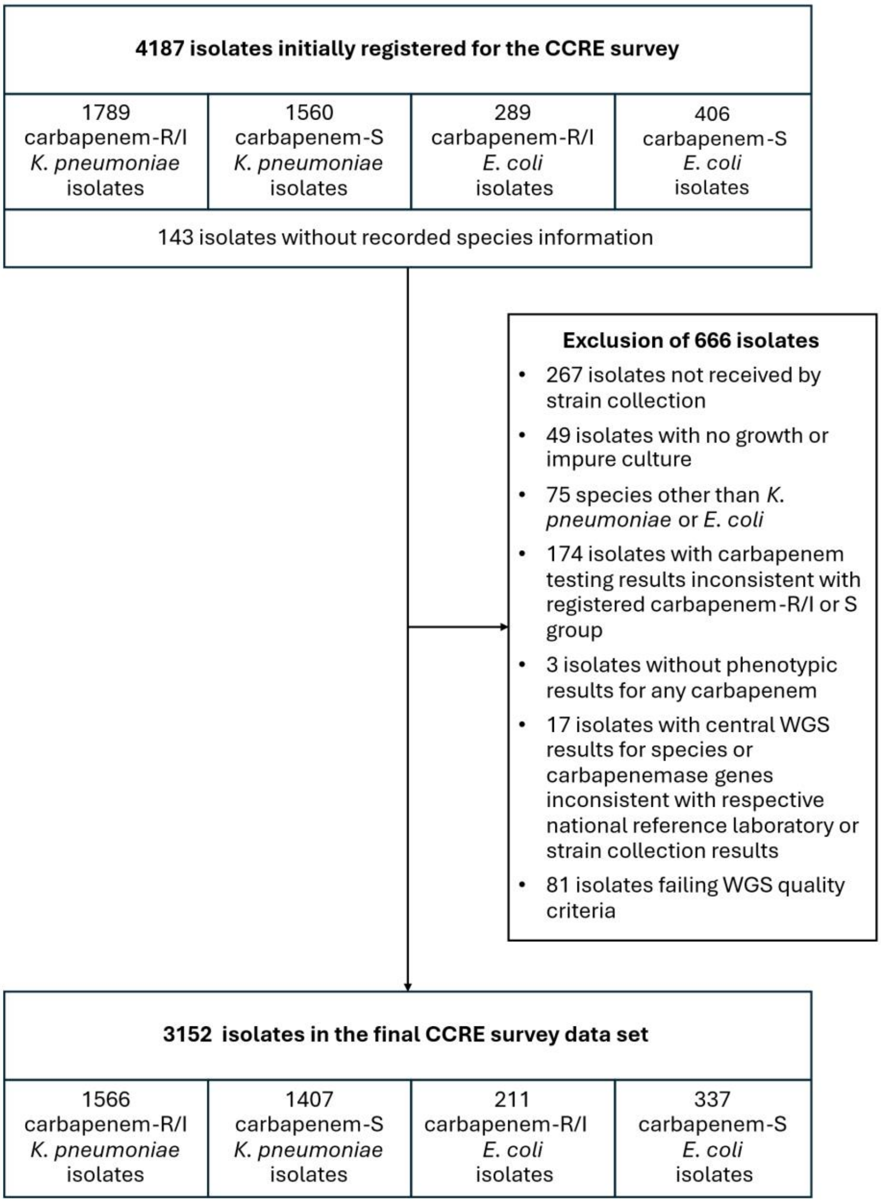

**The analysis of the* K. pneumoniae SC *isolates is described in a separate manuscript.*

CCRE survey, survey of carbapenem-and/or colistin-resistant Enterobacterales; R, resistant; I, susceptible, increased exposure; S, susceptible; WGS, whole genome sequencing.

Figure A2 Percentage of 211 carbapenem-R/I and 337 carbapenem-S *Escherichia coli* isolates from the carbapenem- and/or colistin-resistant Enterobacterales (CCRE) survey with phenotypic antimicrobial susceptibility testing results

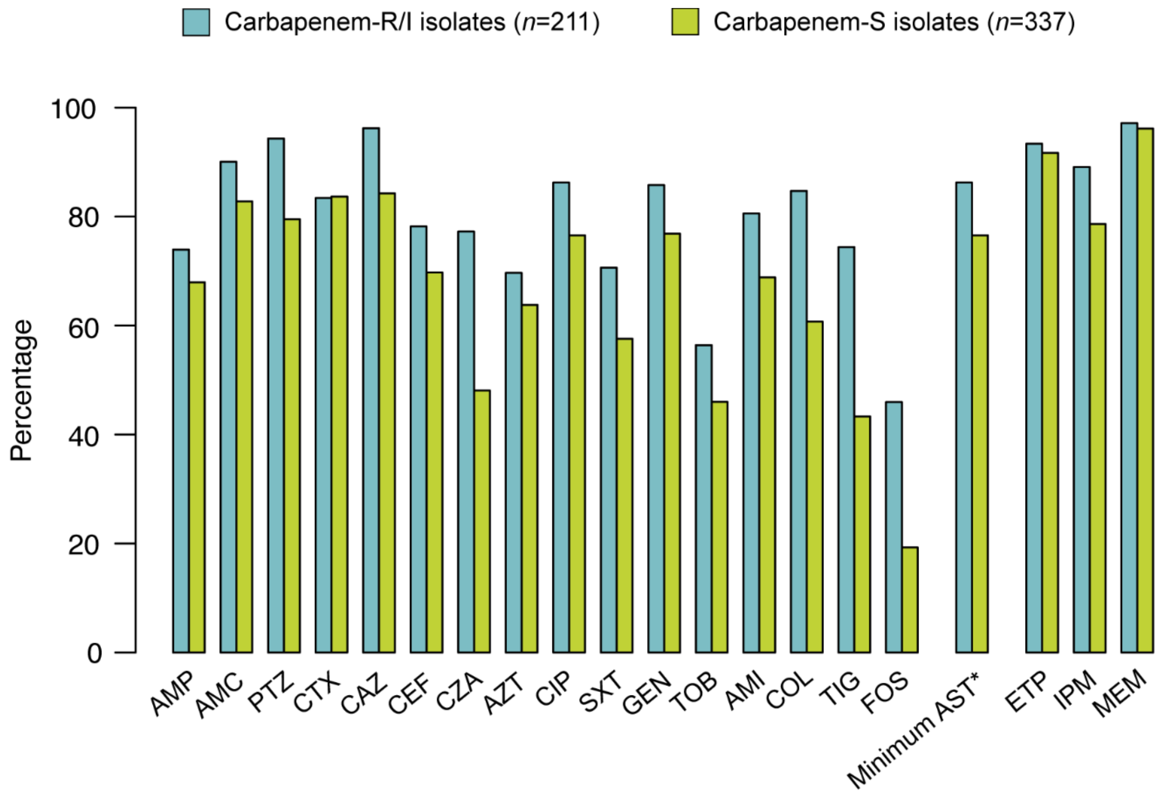

R, resistant; I, susceptible, increased exposure; S, susceptible.
*Abbreviations: AMP – ampicillin, AMC – amoxicillin-clavulanic acid, PTZ – piperacillin-tazobactam, CTX – cefotaxime, CAZ – ceftazidime, CEF – cefepime, CZA – ceftazidime-avibactam, AZT - aztreonam, CIP – ciprofloxacin, SXT – trimethoprim-sulfamethoxazole, GEN – gentamicin, TOB – tobramycin, AMI – amikacin, COL – colistin, TIG – tigecycline, FOS – fosfomycin, ETP – ertapenem, IPM – imipenem, MEM – meropenem. *Minimum antimicrobial susceptibility testing (AST) is defined as AST performed for ≥1 carbapenem, ≥1 extended-spectrum cephalosporin and ≥1 fluoroquinolone.*

Figure A3 Country distribution of major sequence types among 211 carbapenem-R/I *Escherichia coli* isolates from the carbapenem- and/or colistin-resistant Enterobacterales (CCRE) survey

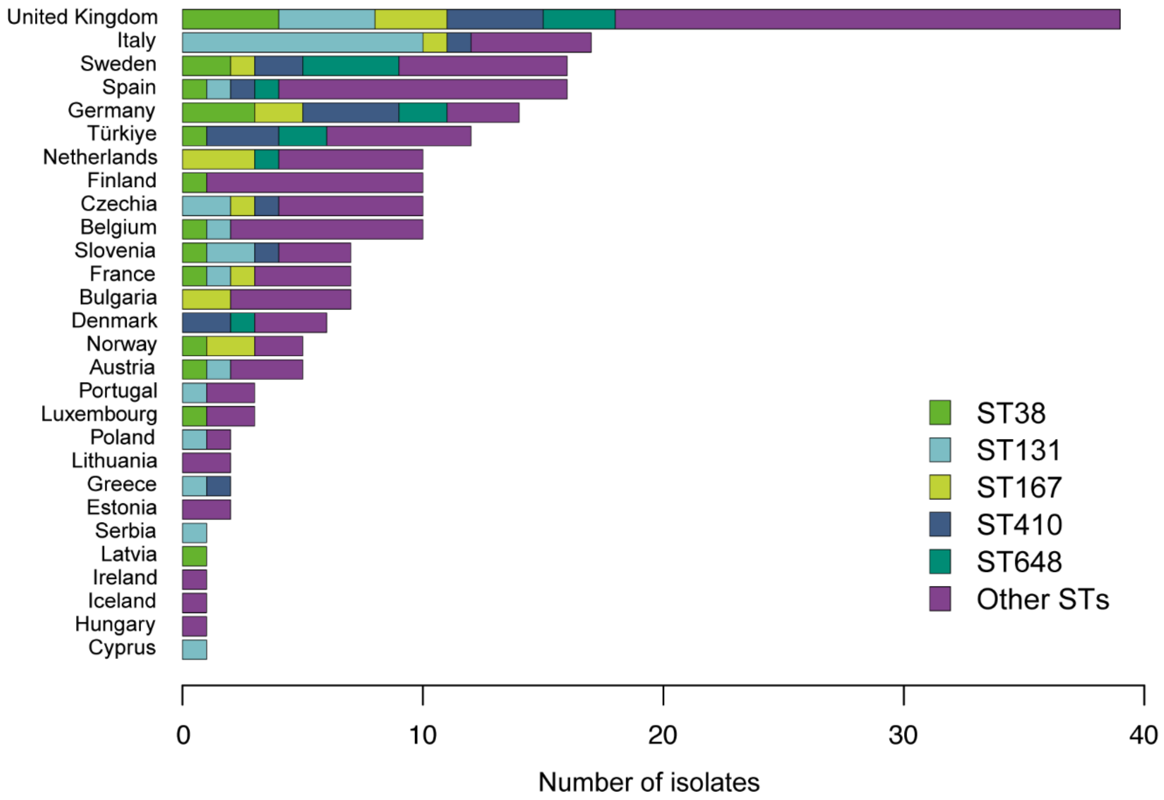

ST, sequence type.

Figure A4 SNP differences between *Escherichia coli* isolates from the carbapenem- and/or colistin-resistant Enterobacterales (CCRE) survey and their nearest neighbours from the same hospital (A-C), a different hospital in the same country (D-F) and a different country (G-I)

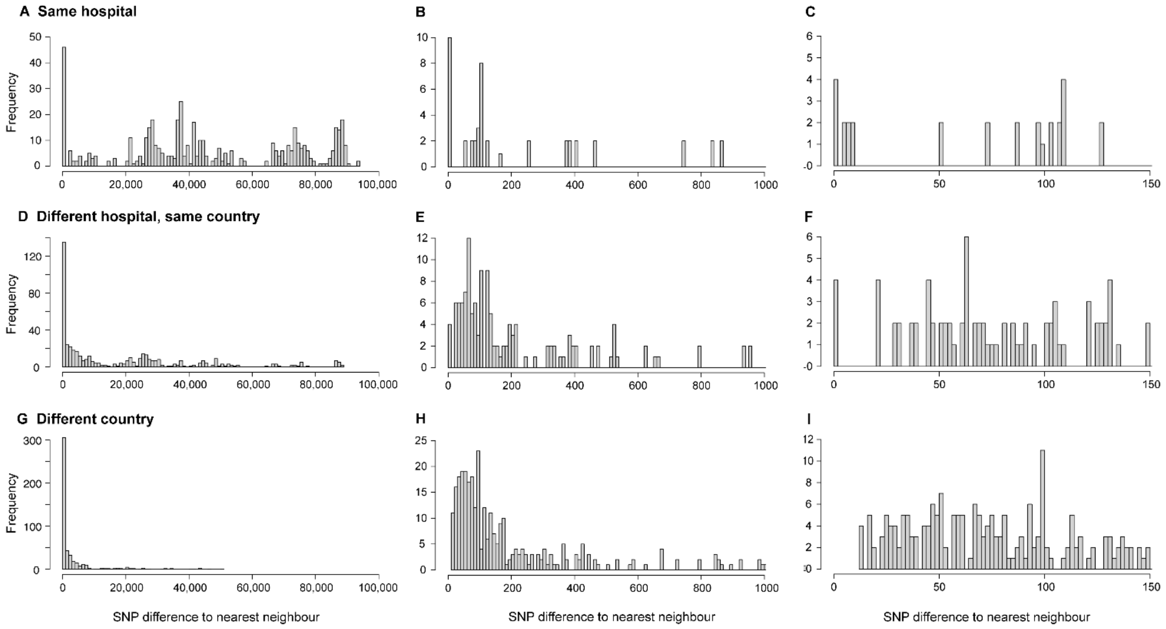

The histograms show the full range of pairwise single nucleotide polymorphism (SNP) distances on the left, up to 1000 SNPs in the middle, and up to 150 SNPs on the right. Note also the different scales on the y-axes. A total of 493 isolates were used in this analysis after the exclusion of 55 isolates where duplicate isolates from the same patient could not be excluded. Exclusion was based on either the absence of patient age/sex information (31 isolates) or matching age/sex profiles among same-hospital isolates (24 isolates). Nearest-neighbour SNP distances are shown for 469 isolates in A-C that are from hospitals that submitted ≥2 isolates, 466 isolates in D-F that are from countries with ≥2 contributing hospitals, and for all 493 isolates in G-I.

Figure A5 Distribution of carbapenemase genes detected in 211 carbapenem-R/I *Escherichia coli* isolates from the carbapenem- and/or colistin-resistant Enterobacterales (CCRE) survey, by country

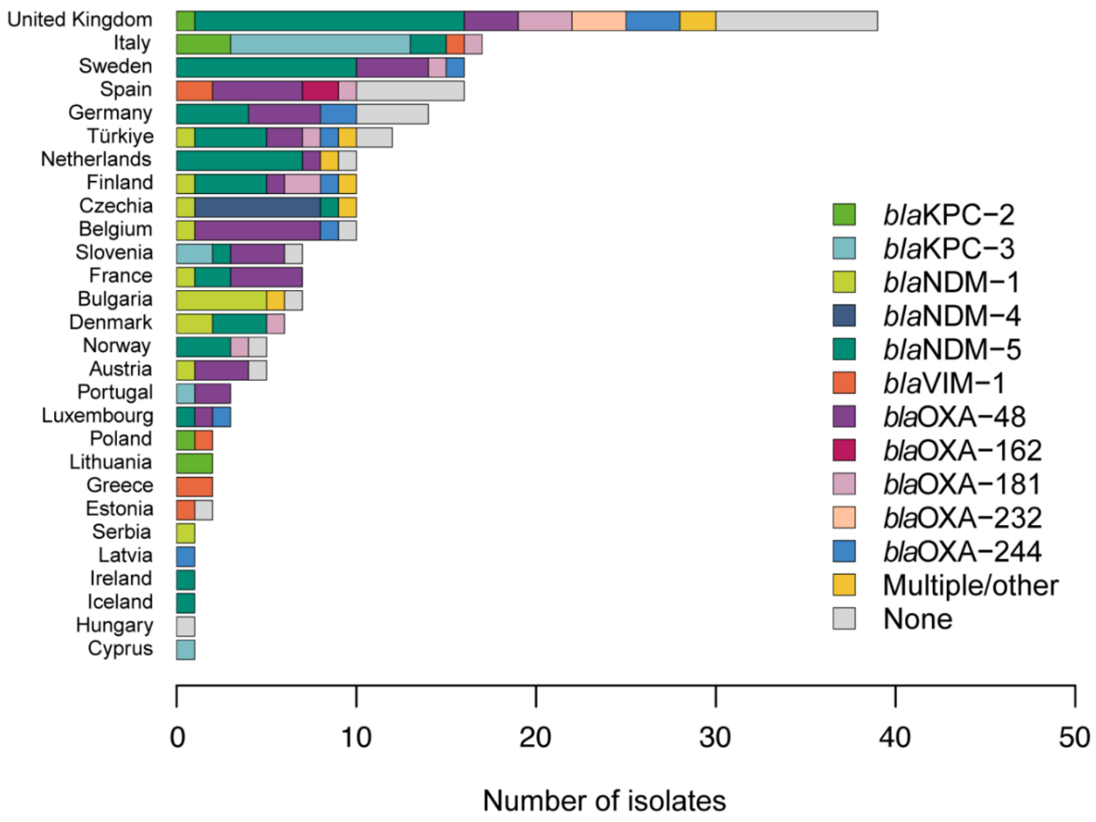

Genes comprising ≤1% of all carbapenemase genes are included in the "Multiple/other" category.
R, resistant; I, susceptible, increased exposure.

Figure A6 Combinations of resistance genes and mutations among (A) 211 carbapenem-R/I and (B) 337 carbapenem-S *Escherichia coli* isolates from the carbapenem- and/or colistin-resistant Enterobacterales (CCRE) survey

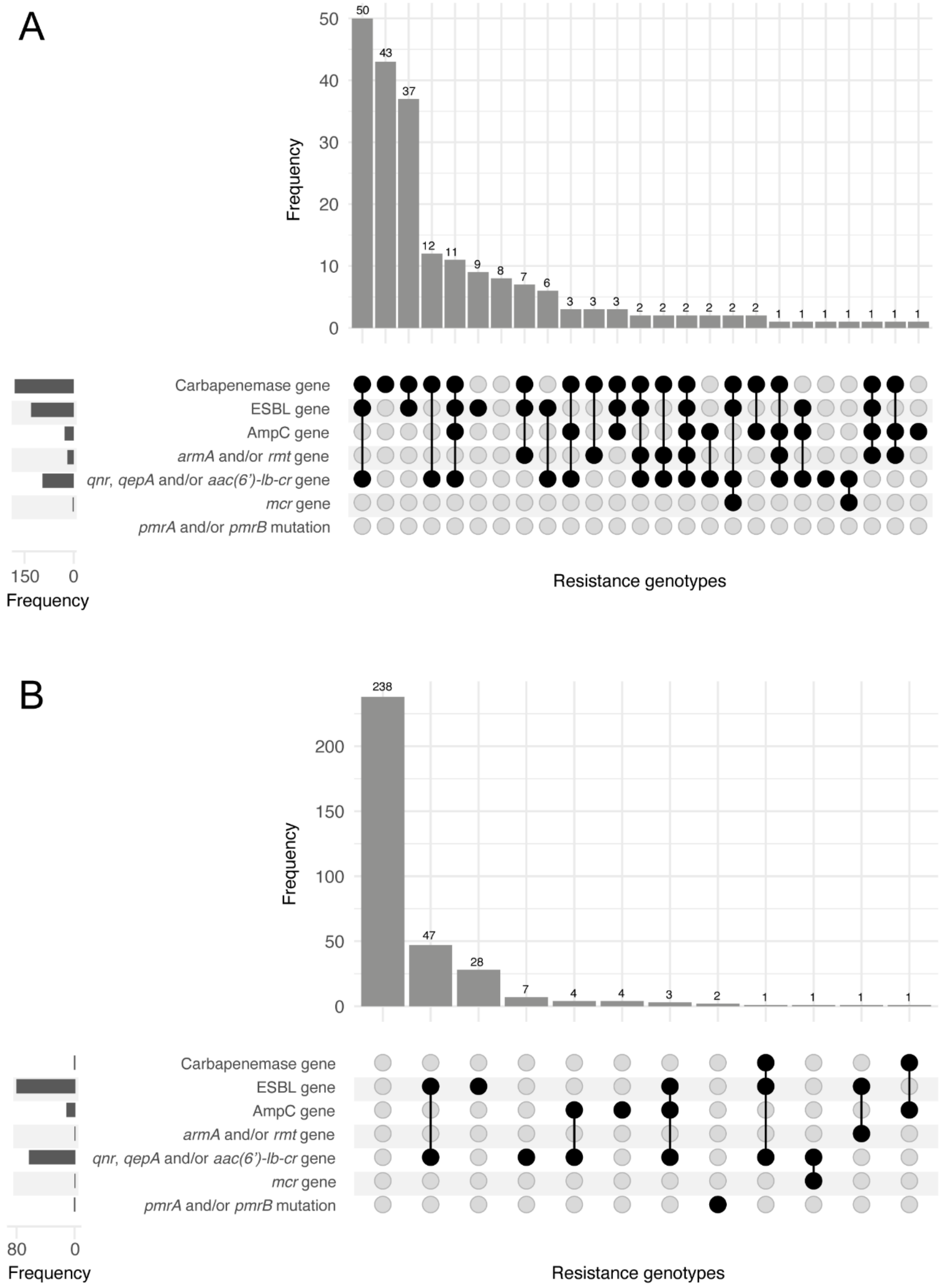

R, resistant; I, susceptible, increased exposure; S, susceptible, ESBL, extended-spectrum beta-lactamase. Frequencies of all combinations are shown in the upper bar plot while frequencies of individual genes/mutations are shown on the left.

Figure A7 Phylogenetic trees of *Escherichia coli* isolates belonging to (A) ST361 (n=374), (B) ST405 (n=974), (C) ST410 (n=1182) and (D) ST648 (n=789)

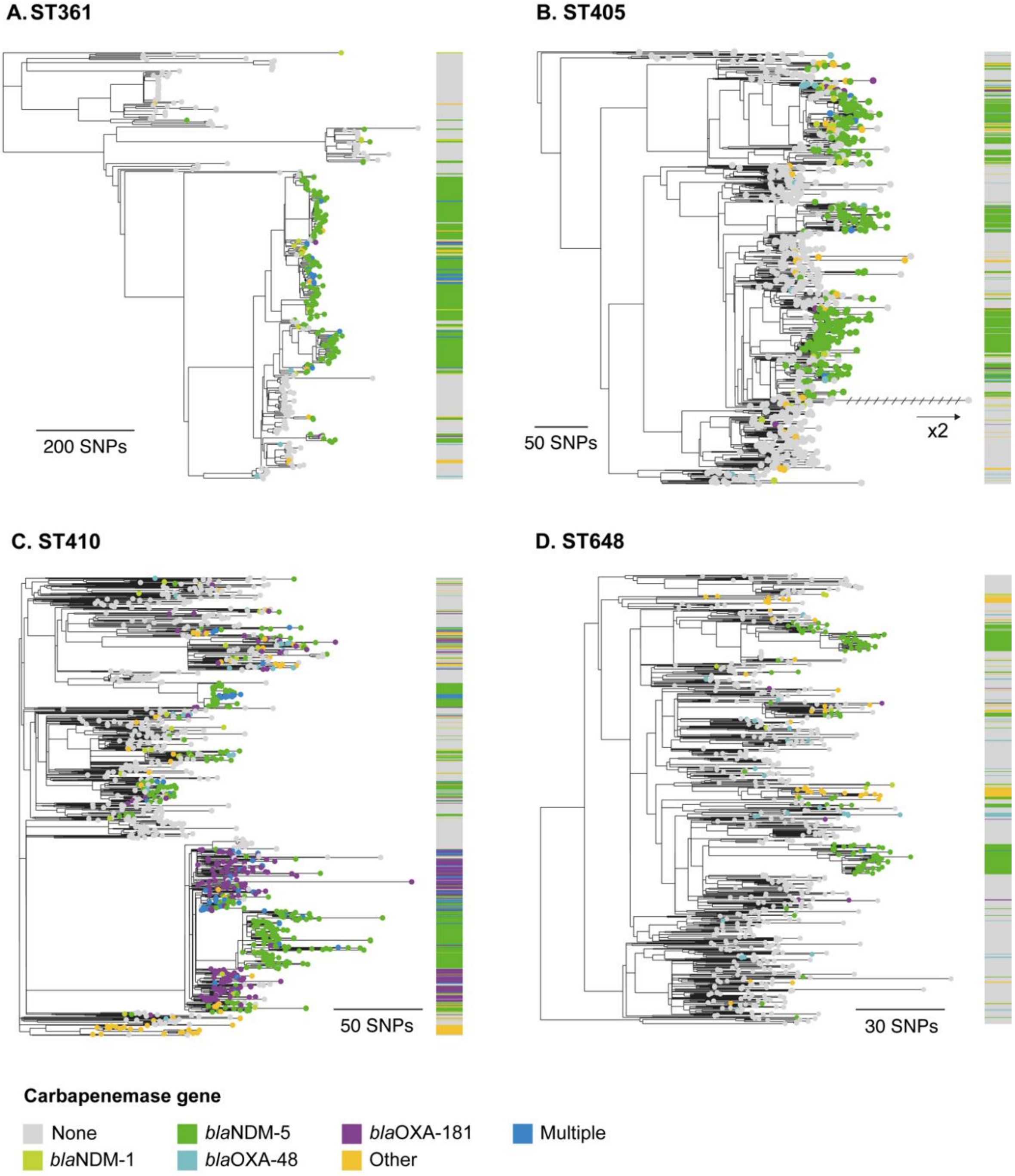

The phylogenetic trees (and above stated numbers) include a small number of isolates from related sequence types (STs) that are nested among the main STs. The illustrated trees of ST405, ST410 and ST648 represent subtrees of the full phylogenetic trees that are available using the Microreact URLs below. One branch in (B) has been shortened for visualisation purposes. The isolate tips and metadata columns are coloured by the carbapenemase gene variants. The scale bars represent the number of SNPs. Interactive versions of these trees with additional metadata and genotyping data are available at: https://microreact.org/project/ecoli-st361-ccre-survey (ST361), https://microreact.org/project/ecoli-st405-ccre-survey (ST405), https://microreact.org/project/ecoli-st410-ccre-survey (ST410), https://microreact.org/project/ecoli-st648-ccre-survey (ST648).
